# Indirect comparison of efficacy and safety between immune checkpoint inhibitors and antiangiogenic therapy in advanced non–small-cell lung cancer

**DOI:** 10.1038/s41598-018-27994-x

**Published:** 2018-06-26

**Authors:** Jin-Hua Chen, Jia-Lian Yang, Che-Yi Chou, Jiun-Yi Wang, Chin-Chuan Hung

**Affiliations:** 1 Graduate Institute of Data Science, College of Management, Taipei Medical University, Taipei, Taiwan Republic of China; 2 Research Center of Biostatistics, Taipei Medical University, Taipei, Taiwan Republic of China; 3 Department of Pharmacy, College of Pharmacy, China Medical University, 91 Hsueh-Shih Road, Taichung, 40402 Taiwan Republic of China; 4 Kidney Institute and Division of Nephrology, Department of Internal Medicine, China Medical University Hospital, 2 Yude Road, Taichung, 40447 Taiwan Republic of China; 5 Department of Healthcare Administration, Asia University, Wufeng, Taichung, 41354 Taiwan Republic of China; 6 Department of Pharmacy, China Medical University Hospital, 2 Yude Road, Taichung, 40447 Taiwan Republic of China

## Abstract

In this study, we conducted an indirect comparison analysis to compare the efficacy and safety of immune checkpoint inhibitors with those of antiangiogenic therapy—two effective treatment methods for advanced non–small-cell lung cancer (NSCLC). Eligible randomised control trials of immune checkpoint inhibitors, antiangiogenic therapy, and doublet platinum-based therapy published up to July 2017 were comprehensively analysed. Through the indirect comparison analysis of 37 trials involving 16810 patients, treatments were compared for overall survival (OS) and grade 3–5 adverse events. For first-line treatment, the use of pembrolizumab alone (hazard ratio [HR]: 0.6; 95% confidence interval [CI]: 0.4–0.91) and a combination of bevacizumab and doublet platinum-based therapy (HR: 0.86; 95% CI: 0.75–0.99) demonstrated substantial survival benefits compared with doublet platinum-based therapy. For subsequent treatment, nivolumab may provide higher efficacy and lower toxicity than antiangiogenic therapy. Overall, anti-PD1 monoclonal antibodies may be superior to antiangiogenic therapy in terms of OS and grade 3–5 adverse events. This meta-analysis suggests that pembrolizumab and nivolumab might be favourable choices for first-line and subsequent treatment, respectively, for patients with advanced NSCLC. Additional randomised control trials are required for a comprehensive evaluation of the outcomes among regimens.

## Introduction

Lung cancer is one of the most common cancers worldwide and is the leading cause of cancer-related mortality^1^. Non–small cell lung cancer (NSCLC) accounts for 85% of lung cancer cases, and advanced NSCLC has a 5-year survival rate of 4%^2^. Recent advances include targeting mutations such as EGFR, ALK, and ROS1; however, only a small proportion (<20%) of patients with advanced NSCLC carry these mutations and thus benefit from target therapies^3,4^. The vast majority of patients with advanced NSCLC are administered conventional chemotherapy and radiotherapy, with only a modest improvement in survival rate. Therefore, the development of better treatment options is imperative to improve the survival and quality of life of patients with advanced NSCLC.

Recently, immunotherapy, which aims to enhance the immune response towards tumours, has been a promising treatment for patients with advanced NSCLC^5–13^. Immune checkpoint inhibitors have been demonstrated to be effective in the treatment of various malignancies^14^. Immune checkpoints are one of the defence mechanisms in human immunity. Higher levels of immune checkpoint receptors, such as programmed cell death protein 1 (PD-1) and cytotoxic lymphocyte-associated antigen 4 (CTLA-4), are expressed when T cells are activated^15–17^. The NSCLC cells may express PD-1 ligand (PD-L1) that bind to PD-1 and suppress the activity of T cells. Therefore, anti-PD-1 antibodies binding to PD-1 receptors would inhibit the function of T cells and efficiently strengthen immunity against tumour cells^18^. The results of several randomised controlled trials (RCTs) have demonstrated that anti-PD-1/PD-L1 antibodies, such as pembrolizumab, nivolumab, and atezolizumab, improve survival in patients with advanced NSCLC^19–22^, thus providing a new treatment choice.

Antiangiogenesis is another promising treatment option for patients with advanced NSCLC. Tumour angiogenesis is driven by vascular endothelial growth factor (VEGF), an essential factor for tumour progression^23^. Under hypoxia stress, VEGF is secreted by tumour cells, which binds to VEGF receptor 2 (VEGFR2) on endothelial cells. Consequently, tumour cell proliferation is promoted and angiogenic growth is initiated. The increased tumour vascularity supplies tumours with sufficient nutrients and oxygen for cancer cell proliferation. Currently, VEGF is a crucial therapeutic target for cancer treatment. The development of antiangiogenic agents has mainly focused on two approaches: monoclonal antibodies directed against either VEGF or VEGFR, and small molecule tyrosine kinase inhibitors (TKIs) targeting the kinase domain of the VEGFR. The Food and Drug Administration of the United States has approved two antiangiogenic monoclonal antibodies for NSCLC treatment, namely anti-VEGF antibody bevacizumab and anti-VEGFR2 antibody ramucirumab^24–26^. The marked early responses of antiangiogenic TKIs directed against VEGFR2 has resulted in the design of many structurally related antiangiogenic TKIs^27–29^. Several RCTs have been conducted; however, the benefits of antiangiogenic TKIs for patients with advanced NSCLC remain uncertain.

Several studies have demonstrated that both immune checkpoint inhibitors and antiangiogenic therapy are effective against advanced NSCLC in considerably improving the progression-free survival and overall survival (OS). Additionally, the use of immune checkpoint inhibitors and antiangiogenic therapy has been reported to increase the objective response rates relative to standard chemotherapy^30,31^. Because of the lack of head-to-head RCTs comparing immune checkpoint inhibitors and antiangiogenic therapy, the most favourable treatment for first-line or subsequent therapy in advanced NSCLC remains unclear. Indirect comparison is a biostatistical approach to indirectly compare the relative effects between regimens without a direct comparison. To investigate the role of immune checkpoint inhibitors and antiangiogenic therapy in advanced NSCLC, we performed an indirect comparison to compare the safety and efficacy of immune checkpoint inhibitors, antiangiogenic therapy, and conventional chemotherapy.

## Methods

### Literature search and study selection

The protocol was registered and approved in Prospective Register of Systematic Reviews, PROSPERO (CRD42016051388).

Cochrane Central Register of Controlled Trials (CENTRAL), Web of science, EMBASE and Medline were searched for eligible randomized controlled trials up to July 2017. The meeting abstracts from the American Society of Clinical Oncology (ASCO) and European Society for Medical Oncology (ESMO) were also searched for eligible trials. In addition, the US National Institutes of Health Ongoing Trials Register (Clinicaltrial.gov) was searched for unpublished data of randomized controlled trials. The search algorithm consisted of the medical subject headings (MeSH) and text words for non-small cell lung cancer and each treatment options were as following: non-small cell lung cancer (NSCLC) AND antiangiogenesis OR sorafenib OR ramucirumab OR bevacizumab OR vandetanib OR sunitinib OR nintedanib OR pazopanib OR everolimus OR PD-1 OR PD-L1 OR nivolumab OR CTLA-4 OR pembrolizumab OR atenolizumab OR cisplatin OR carboplatin OR docetaxel OR pemetrexed AND clinical trial.

The following inclusion criteria were required for eligible randomized controlled trials: (1) prospective randomized controlled trials reporting on efficacy and toxicity; (2) enrolled patients with unresectable locally advanced or metastatic NSCLC either treatment-naive or first-line chemotherapy failure; (3) treated with anti-angiogenesis inhibitors, immunotherapy or chemotherapy as first-line therapy or subsequent therapy; (4) the performance scores of enrolled patients were less than 2 and aged from 18 to 75 years old with adequate hematological, renal and liver function; (5) language limited to English or Chinese; (6) treatments did not contain erlotinib, gefitinib, afatinib and cetuxiamb. The abstracts and full text were independently evaluated by two reviewers and discussed with a third author if disagreements occurred.

### Data extraction and risk of bias assessment

The primary endpoint was overall survival. The secondary endpoints were progression free survival and all grade 3 to 5 adverse events (according to National Cancer Institute Common Terminology Criteria for Adverse Events version 4.0). The hazard ratios (HRs) with 95% confidence intervals (CIs) of progression free survival and overall survival were extracted for estimating effects. As for all grade 3 to 5 adverse events, number of patients and the number of total events were extracted for evaluation. The patient’s characteristics, dosing regimen, study design and follow up time were also collected. Cochrane risk of bias tool (version 5.1.0) was used to assess the risk of bias of included trials and the evaluated items were scored as low, high, or unknown risk of bias. The data extraction and risk of bias assessment were independently performed by two reviewers and a third author handled with conflicts.

### Statistical Analysis

This systemic review and indirect comparison were performed with frequentist model and reported according to the preferred reporting items for systematic reviews and meta-analyses (PRISMA) guidelines (Supplementary Table S1). For the efficacy evaluation, the outcome measures of overall survival and progression free survival were the hazard ratios (HRs) with 95% CIs. For toxicity, the outcome measures of all grade 3 to 5 toxicities were odds ratios (ORs) with 95% CIs. The primary and secondary outcomes of first-line therapy and subsequent therapy were analyzed separately to reduce the heterogeneity.

For direct meta-analysis, odds ratio and hazard ratios were pooled by using a DerSimonian-Laird random-effects model in Revman 5.3. (Cochrane collaboration, Copenhagen: The Nordic Cochrane Centre, The Cochrane Collaboration, 2014.). This method was based on the inverse-variance approach which study weight was adjusted based on extent of variation among the intervention effects. Mantel-Haenszel method was used to calculate the odds ratio. On the other hand, the hazard ratio was estimated with inverse-variance method. Next, an indirect comparison was conducted using frequentist model^32,33^. The important assumption of indirect comparison is transitivity^34,35^. With this assumption, estimated effect of intervention A versus intervention B can be obtained via intervention C, if the information of intervention A versus intervention C and intervention B versus intervention C were available^36^. The key concept of transitivity contained that: (1) patients in A-B of the direct comparison studies are similar from those in B-C and A-C studies, included patients can randomly be allocated to any of intervention being compared indirectly; (2) the direct comparisons between interventions were not different about the distribution of effect modifiers; (3) the common intervention was similar in different trials^34,37^. To evaluate the appropriateness of conducting indirect comparison, we conducted meta-regression and its results showed interventions were not different about the distribution of effect modifiers in this study^38^. ORs and HRs were pooled by random-effects model and conducted in Frequentist framework by STATA 13.1 (StataCorp, College Station, TX, USA) and R software 3.31 (R Core Team (2013). R: A language and environment for statistical computing. R Foundation for Statistical Computing, Vienna, Austria. http://www.R-project.org/) with netmeta package (https://CRAN.R-project.org/package=netmeta). Direct and indirect treatment effects were merged into a single effect size and the relative effects between interventions were presented as ORs and HRs with 95% CIs.

To evaluate the quality of results, heterogeneity analysis, and sensitivity analysis were performed. For pair-wise and indirect meta-analysis, Cochran Q test and the *I*² statistic were used to evaluate the heterogeneity across the studies. Heterogeneity existed with *P*-value lower than 0.1 in Cochran Q test^39^. Subgroup analysis and sensitivity analysis were conducted if there was heterogeneity. Sensitivity analysis was used to assess the potential bias in indirect comparison by excluding high risk studies.

### Data availability

The datasets generated during and/or analyzed during the current study are available from the corresponding author on reasonable request.

## Result

### Search results

**Figure 1:**
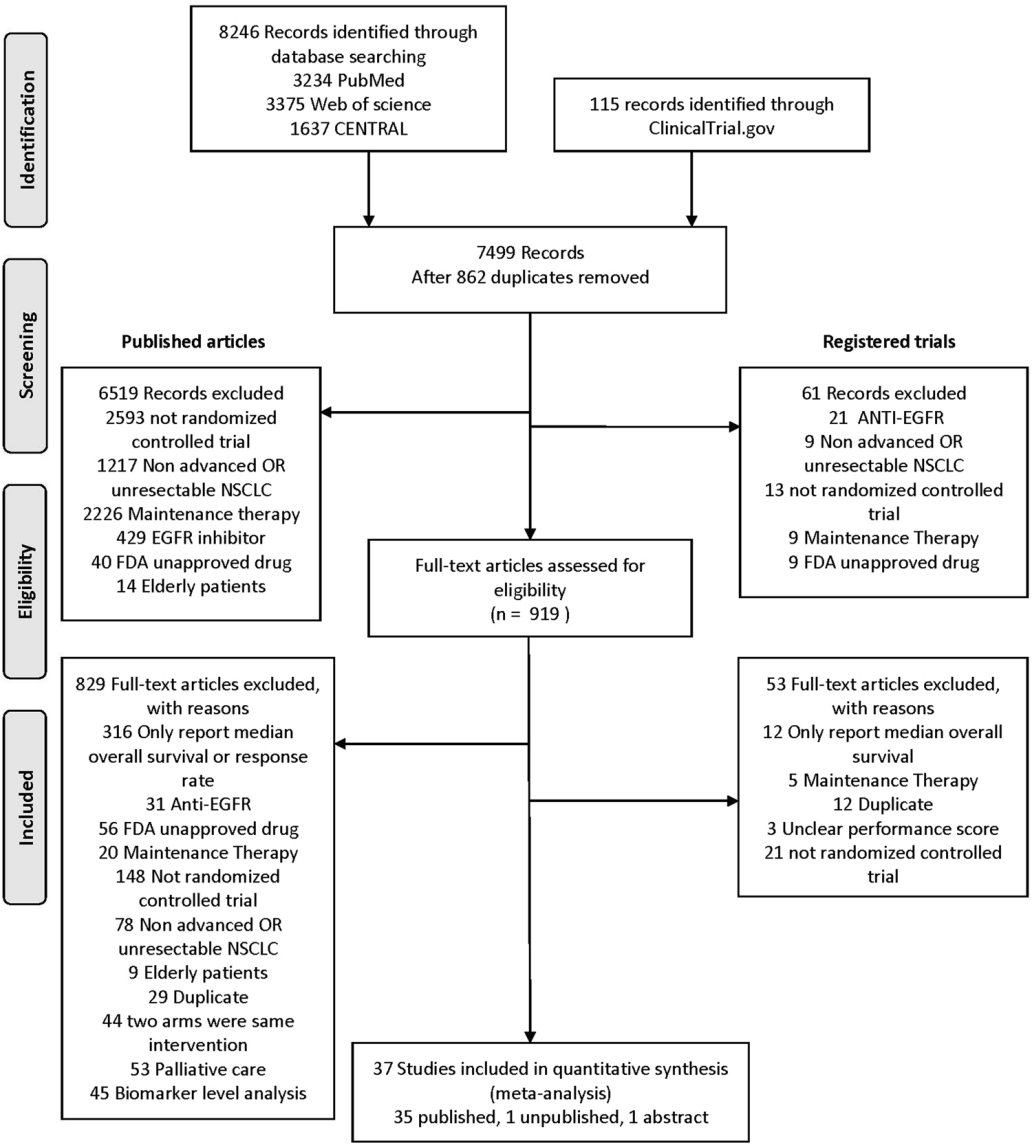
PRISMA flow diagram of randomised control trials identified, included and excluded.

A total of 919 studies were evaluated by two independent reviewers and 37 RCTs involving 16810 patients were included to conduct meta-analysis and indirect comparisons (Fig. 1). The characteristics of included trials were showed in Supplementary Table S2. Eighteen trials were conducted as first line setting and nineteen trials were designed as subsequent therapy. Among the trials of first line setting, eighteen trials compared anti-angiogenetic agents or immune checkpoint inhibitors with doublet platinum-based treatment^25,40–56^. In terms of the trials of subsequent therapy, seventeen trials compared anti-angiogenic agents or immune checkpoint inhibitors with docetaxel^20,22,24,57–70^ and two trials compared these newer treatments with pemetrexed^71,72^. Nineteen anticancer agents were analyzed, including anti-angiogenetic agents (bevacizumab, aflibercept, ramucirumab, nintedanib, axitinib, sorafenib, vandetanib, and sunitinib), immune checkpoint inhibitors (ipilimumab, pembrolizumab, nivolumab and atezolizumab) and traditional chemotherapy (cisplatin, carboplatin, oxaliplatin, gemcitabine, paclitaxel, docetaxel and pemetrexed) (Network plot, Supplementary Fig. S1).

### Risk of bias

The quality of the included RCTs were generally good with low risk of bias (Supplementary Fig. S2). The most common bias was the lack of blinding in about 38% of included trials with open-label designed^20,22,40–42,45,47,49,57–59,63,66,72^. In the domain of other risk of bias, one trial by Wang Y. *et al*.^52^ was at high risk of bias due to single center design.

### Overall survival (OS)

The results of pairwise meta-analysis of direct comparisons of OS in first line and subsequent setting were presented in Supplementary Figs S3, S4 and S5. In the first line setting, use of pembrolizumab significantly prolonged OS (HR: 0.60; 95%CI: 0.41–0.88; p = 0.010; heterogeneity: single trial). In the subsequent setting, the use of nivolumab (HR: 0.67; 95%CI: 0.55–0.82; p = 0.0001; heterogeneity: p = 0.24; *I*^2^ = 27%), pembrolizumab (HR: 0.71; 95%CI: 0.58–0.87; p = 0.001; heterogeneity: single trial), atezolizumab (HR: 0.73; 95%CI: 0.63–0.84; p < 0.0001; heterogeneity: p = 1.00; *I*^2^ = 0%) and ramucirumab plus docetaxel (HR: 0.86; 95%CI: 0.75–0.98; p = 0.02; heterogeneity: p = 1.00; *I*^2^ = 0%) showed significant OS benefit versus standard chemotherapy.

An indirect comparison was performed to compare the OS of first line and subsequent setting included in the supplementary Fig. S1. For the first line setting, both use of pembrolizumab alone (HR: 0.6; 95%CI: 0.4–0.91) and the combination of bevacizumab and doublet platinum-base therapy (HR: 0.86; 95%CI: 0.75–0.99) showed significant survival benefit as compared to doublet platinum therapy. Overall, anti-PD1 monoclonal antibodies appears superior to anti-angiogenic therapies in terms of OS (Table 1). The use of pembrolizumab alone was associated with statistically significant survival benefit as compared to the combination of axitinib and doublet platinum-based therapy (HR: 0.41; 95%CI: 0.22–0.78), the combination of sorafenib and doublet platinum-based therapy (HR: 0.57; 95%CI: 0.36–0.89), and the combination of vandetanib and doublet platinum-based therapy (HR: 0.52; 95%CI: 0.28–0.96); it was also superior to the combination of ramucirumab and doublet platinum-based therapy (HR: 0.58; 95%CI: 0.32–1.05) and the combination of bevacizumab and doublet platinum-based therapy (HR: 0.69; 95%CI: 0.45–1.07), although these difference did not reach statistical significance. In addition, the use of pembrolizumab alone resulted in significant survival advantage when compared to nivolumab alone, regardless of PD-1/PD-L1 expression level (HR: 0.59; 95%CI: 0.36–0.97).

**Table 1:** Indirect comparison for overall survival in first line therapy.

Axitinib + PLA	1.68 (1.02,2.76)*	1.56 (0.93,2.6)	1.42 (0.81,2.48)	**2.41** (1.28,4.55)*	1.61 (0.65,4.01)	0.86 (0.51,1.47)	1.45 (0.9,2.34)	1.41 (0.75,2.65)	1.37 (0.83,2.28)	1.26 (0.65,2.44)
	Bevacizumab + PLA	0.93 (0.74,1.17)	0.85 (0.62,1.16)	1.44 (0.93,2.23)	0.96 (0.44,2.11)	**0.52** (0.39,0.68)*	**0.86** (0.75,0.99)*	0.84 (0.54,1.3)	0.82 (0.66,1.01)	0.75 (0.47,1.21)
		Ipilimumab + PLA	0.91 (0.65,1.28)	1.55 (0.99,2.44)	1.03 (0.47,2.3)	**0.56** (0.41,0.75)*	0.93 (0.77,1.12)	0.9 (0.57,1.42)	0.88 (0.69,1.13)	0.81 (0.5,1.32)
			Nivolumab	**1.7** (1.03,2.81)*	1.13 (0.5,2.59)	**0.61** (0.42,0.88)*	1.02 (0.77,1.35)	0.99 (0.6,1.64)	0.97 (0.7,1.34)	0.89 (0.52,1.51)
				Pembrolizumab	0.67 (0.28,1.61)	**0.36** (0.22,0.58)*	**0.6** (0.4,0.91)*	0.58 (0.32,1.05)	**0.57** (0.36,0.89)*	**0.52** (0.28,0.96)*
					Pembrolizumab + PLA	0.54 (0.24,1.21)	0.9 (0.41,1.96)	0.87 (0.36,2.11)	0.85 (0.39,1.89)	0.78 (0.32,1.92)
						Pemetrexed	**1.68** (1.32,2.12)*	**1.63** (1.01,2.62)*	**1.59** (1.2,2.12)*	1.46 (0.87,2.43)
							PLA	0.97 (0.64,1.47)	0.95 (0.81,1.12)	0.87 (0.55,1.37)
								Ramucirumab + PLA	0.98 (0.63,1.53)	0.9 (0.48,1.66)
									Sorafenib + PLA	0.92 (0.57,1.48)
										Vandetanib + PLA

The hazard ratio (HR) with 95% confidence interval for a given comparison was read in the intersection of two treatments. *P < 0.05. PLA: doublet platinum-based treatment.

In the subsequent setting, the single use of anti-PD1/PD-L1 monoclonal antibodies (atezolizumab alone, pembrolizumab alone and nivolumab alone) showed significant survival benefit as compared to docetaxel or pemetrexed (Table 2). The combination of ramucirumab and docetaxel also resulted in survival advantage when compared to docetaxel (HR: 0.79; 95% CI: 0.64–0.98). Overall, in the subsequent setting, the single use of anti-PD1/PD-L1 monoclonal antibodies appears superior to anti-angiogenic therapies in terms of OS (Table 2). The use of nivolumab alone was associated with statistically significant survival benefit as compared to the combination of ramucirumab and docetaxel (HR: 0.79; 95%CI: 0.64–0.98), the combination of sunitinib and pemetrexed (HR: 0.49; 95%CI: 0.31–0.78), and the combination of vandetanib and docetaxel (HR: 0.72; 95%CI: 0.58–0.88); the use of pembrolizumab alone (HR: 0.83; 95%CI: 0.65–1.05) and atezolizumab alone (HR: 0.85; 95%CI: 0.7–1.03) were both superior to the combination of ramucirumab and docetaxel, although the difference were not statistically significant.

**Table 2:** Indirect comparison for overall survival in subsequent therapy.

Aflibercept + Docetaxel	**1.38** (1.12,1.71)*	1.01 (0.87,1.17)	1.07 (0.89,1.3)	1.01 (0.78,1.32)	**1.48** (1.18,1.86)*	**1.42** (1.1,1.84)*	1.02 (0.84,1.25)	1.16 (0.95,1.42)	1.17 (0.96,1.43)	0.73 (0.46,1.15)	1.07 (0.88,1.29)
	Atezoli-zumab	**0.73** (0.63,0.84)*	**0.78** (0.64,0.94)*	**0.73** (0.56,0.95)*	1.07 (0.86,1.34)	1.03 (0.8,1.33)	**0.74** (0.61,0.9)*	0.84 (0.69,1.02)	0.85 (0.7,1.03)	**0.53** (0.34,0.83)*	**0.77** (0.64,0.93)*
		Doce-taxel	1.06 (0.95,1.2)	1 (0.81,1.25)	**1.47** (1.24,1.74)*	**1.41** (1.14,1.73)*	1.01 (0.89,1.16)	1.15 (1,1.32)	**1.16** (1.02,1.32)*	0.72 (0.47,1.11)	1.05 (0.94,1.19)
			Nintedanib + Docetaxel	0.94 (0.74,1.21)	**1.38** (1.13,1.7)*	**1.32** (1.04,1.68)*	0.95 (0.8,1.14)	1.08 (0.9,1.29)	1.09 (0.92,1.3)	0.68 (0.44,1.06)	0.99 (0.84,1.17)
				Nintedanib + Pemetrexed	**1.47** (1.11,1.93)*	**1.4** (1.04,1.9)*	1.01 (0.85,1.2)	1.15 (0.91,1.44)	1.16 (0.9,1.49)	0.72 (0.46,1.12)	1.05 (0.82,1.35)
					Nivo-lumab	0.96 (0.73,1.25)	**0.69** (0.56,0.85)*	**0.78** (0.63,0.97)*	**0.79** (0.64,0.98)*	**0.49** (0.31,0.78)*	**0.72** (0.58,0.88)*
						Pembro-plizumab	**0.72** (0.56,0.92)*	0.82 (0.64,1.05)	0.83 (0.65,1.05)	**0.51** (0.32,0.83)*	**0.75** (0.59,0.95)*
							Peme-trexed	1.13 (0.97,1.32)	1.15 (0.96,1.38)	0.71 (0.47,1.08)	1.04 (0.87,1.24)
								PLA	1.01 (0.84,1.22)	**0.63** (0.41,0.97)*	0.92 (0.77,1.1)
									Ramucirumab + Docetaxel	**0.62** (0.4,0.97)*	0.91 (0.76,1.08)
										Sunitinib + Pemetrexed	1.46 (0.93,2.28)
											Vandetanib + Docetaxel

The hazard ratio (HR) with 95% confidence interval for a given comparison was read in the intersection of two treatments. *P < 0.05. PLA: doublet platinum-based treatment.

### Progression free survival (PFS)

The results of pairwise meta-analysis of direct comparisons of PFS in first line and subsequent setting were presented in Supplementary Figs S6–S8. In the first line setting, statistically significant improvement of PFS were shown in the combination of bevacizumab and doublet platinum-based therapy (HR: 0.62; 95%CI: 0.47–0.82; p = 0.0009; heterogeneity: p = 0.0002; *I*^2^ = 84%), the combination of pembrolizumab and doublet platinum-based therapy (HR: 0.53; 95%CI: 0.31–0.91; p = 0.02; heterogeneity: single trial), and pembrolizumab alone (HR: 0.50; 95%CI: 0.37–0.68; p < 0.00001; heterogeneity: single trial) versus standard doublet platinum-based therapy. In the subsequent setting, statistically significant benefit of PFS were shown in the combination of ramucirumab and docetaxel (HR: 0.75; 95%CI: 0.67–0.84; p < 0.00001; heterogeneity: p = 0.65; *I*^2^ = 0%), the combination of nintedanib and docetaxel (HR: 0.79; 95%CI: 0.68–0.92; p = 0.002; heterogeneity: single trial), the combination of aflibercept and docetaxel (HR: 0.82; 95%CI:0.72–0.94; p = 0.004; heterogeneity: single trial), and the combination of vandetanib and docetaxel (HR: 0.78; 95%CI: 0.70–0.87; p < 0.00001; heterogeneity: p = 0.44; *I*^2^ = 0%) versus docetaxel.

An indirect comparison was performed to compare the PFS of first line and subsequent setting included in the Supplementary Fig. S1. In the first line setting, pembrolizumab alone (HR: 0.5; 95%CI: 0.32–0.79) and combination of bevacizumab and doublet platinum-based therapy (HR: 0.64; 95%CI: 0.52–0.78) showed significantly increased efficacy compared with doublet platinum-based therapy (Supplementary Fig. S9a). Overall, pembrolizumab showed increased efficacy compared with anti-angiogenic therapies, although statistical significance did not reach in some comparisons: pembrolizumab vs combination of bevacizumab and doublet platinum-based therapy (HR: 0.79; 95%CI: 0.48–1.3), pembrolizumab vs combination of ramucirumab and doublet platinum-based therapy (HR: 0.67; 95%CI: 0.34–1.32), pembrolizumab vs combination of sorafenib and doublet platinum-based therapy (HR: 0.54; 95%CI: 0.32–0.91), and pembrolizumab vs combination of vandetanib and doublet platinum-based therapy (HR: 0.66; 95%CI: 0.33–1.32) (Supplementary Table S3).

In the subsequent setting, combination of ramucirumab and docetaxel showed significant increased efficacy compared with docetaxel alone in terms of PFS (HR: 0.74; 95%CI: 0.56–0.98) (Supplementary Fig. S9b and Supplementary Table S4). Although the HR appears to be in favor of pembrolizumab alone (HR: 0.88; 95%CI: 0.61–1.28) and nivolumab alone (HR: 0.78; 95%CI: 0.59–1.03) compared with docetaxel alone, the difference were not statistically significant.

### Toxicity

All grade 3 to 5 adverse events were analyzed. Standard pairwise meta-analysis was performed for the same first line and subsequent settings in the OS and PFS comparisons (Supplementary Figs S10–S12). In the first line setting, pembrolizumab alone (OR: 0.32; 95%CI: 0.20–0.51; p < 0.00001; heterogeneity: single trial) and nivolumab alone (OR: 0.21; 95%CI: 0.14–0.31; p < 0.00001; heterogeneity: single trial) were less toxic than doublet platinum-based treatment. Conversely, combination of sorafenib and doublet platinum-based treatment significantly increased toxicity compared to doublet platinum-based treatment (OR: 2.60; 95%CI: 2.07–3.26; p < 0.0001; heterogeneity: p = 0.63; *I*² = 0%). In the subsequent setting, pembrolizumab alone (OR: 0.27; 95%CI: 0.18–0.40; p < 0.00001; heterogeneity: single trial), nivolumab alone (OR: 0.09; 95%CI: 0.05–0.14; p < 0.00001; heterogeneity: p = 0.26; *I*² = 23%), atezolizumab alone (OR: 0.53; 95%CI: 0.43–0.65; p < 0.0001; heterogeneity: p = 0.55; *I*² = 0%), and pemetrexed alone (OR: 0.39; 95%CI: 0.21–0.72; p = 0.003; heterogeneity: single trial) were less toxic than docetaxel alone. On the other hand, combination of ramucirumab and docetaxel (OR: 1.43; 95%CI: 1.11–1.85; p = 0.006; heterogeneity: p = 0.33; *I*² = 0%) or combination of aflibercept and docetaxel (OR: 2.54; 95%CI: 1.93–3.34; p < 0.0001; heterogeneity: single trial) significantly increased toxicity compared to docetaxel alone.

**Figure 2:**
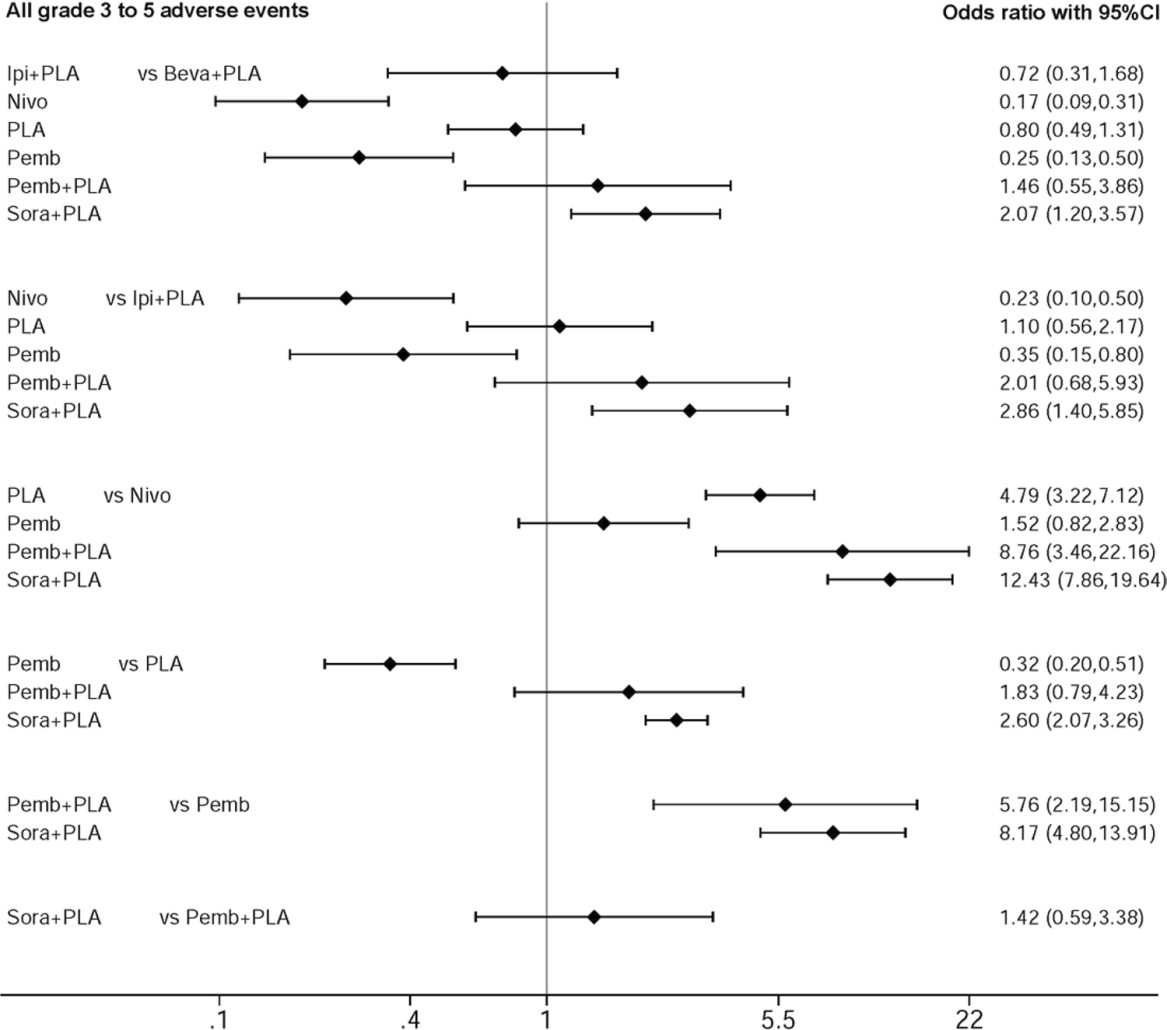
Forest plot of indirect comparison: all grade 3 to 5 adverse events in first line therapy. All individual regimens compared with reference treatment. Odds ratios (OR) and 95% confidence intervals were given. Beva: bevacizumab; Ipi: ipilimumab; Nivo: nivolumab; Pemb: pembrolizumab; Sora: sorafenib; PLA: doublet platinum-based treatment.

**Figure 3:**
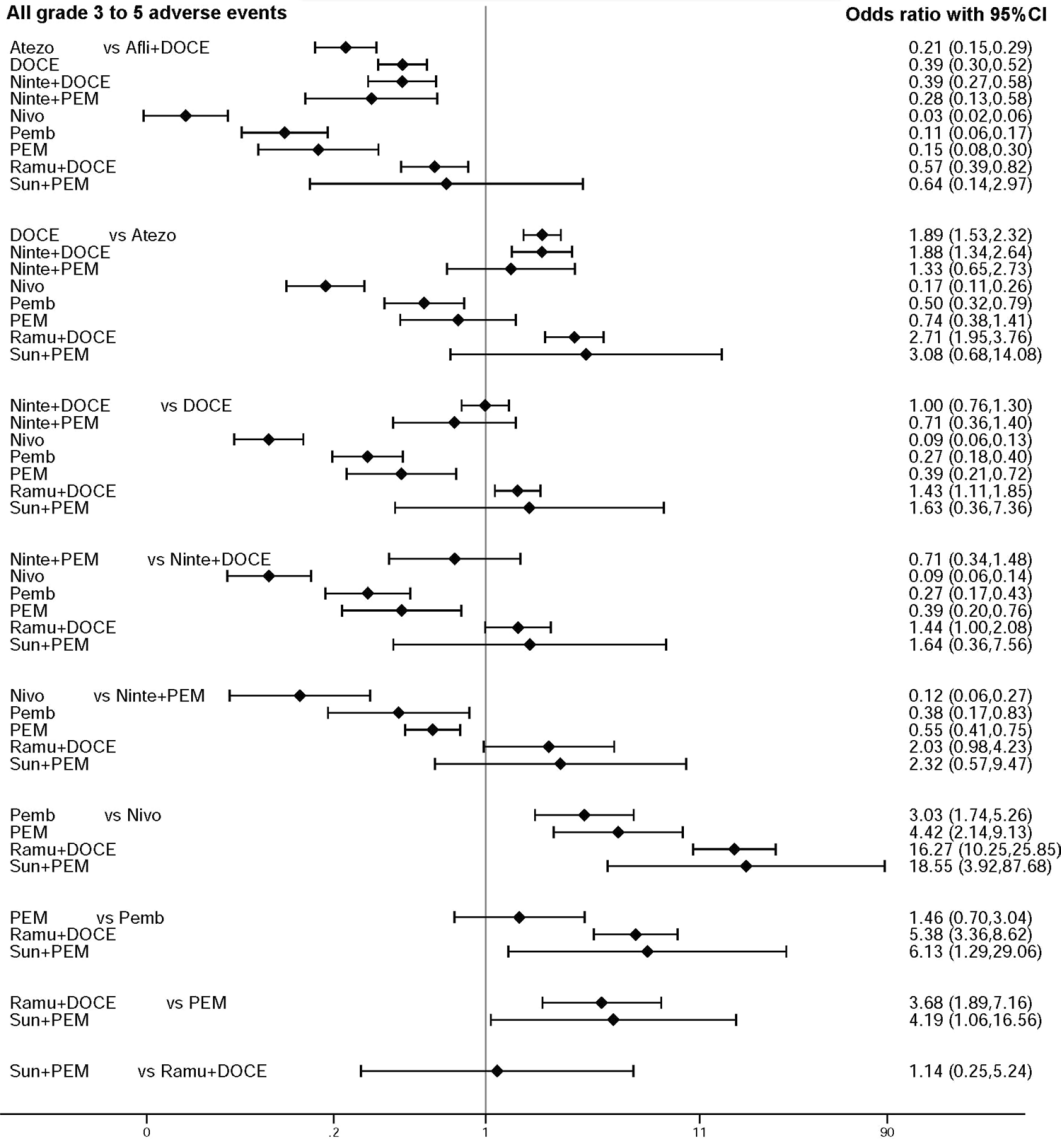
Forest plot of indirect comparison: all grade 3 to 5 adverse events in subsequent therapy. All individual regimens compared with reference treatment. Odds ratios (OR) and 95% confidence intervals were given. Afli: aflibercept; Atezo: atezolizumab; Ninte: nintedanib; DOCE: docetaxel; PEM: pemetrexed; Nivo: nivolumab; Pemb: pembrolizumab; Ramu: ramucirumab; Sun: sunitinib.

Indirect comparison was also performed for all grade 3 to 5 adverse events in first line and subsequent settings (Figs 2 and 3). In the first line setting, anti-PD1 monoclonal antibodies appears to be less toxic than combination of anti-angiogenic agent and doublet platinum-based therapy. Nivolumab alone (OR: 0.17; 95%CI: 0.09–0.31) and pembrolizumab alone (OR: 0.25; 95%CI: 0.13–0.50) significantly decreased toxicity compared to combination of bevacizumab and doublet platinum-based therapy. Anti-PD1 monoclonal antibodies also appears to be less toxic than combination of anti-CTLA4 monoclonal antibody and doublet platinum-based therapy. Nivolumab alone (OR: 0.23; 95%CI: 0.1–0.5) and pembrolizumab alone (OR: 0.35; 95%CI: 0.15–0.80) significantly decreased toxicity compared to combination of ipilimumab and doublet platinum-based therapy. In the first line setting, there was no difference between pembrolizumab alone and nivolumab alone (OR: 1.52; 95%CI: 0.82–2.83) in terms of all grade 3 to 5 adverse events.

In the subsequent setting, combination of anti-angiogenic agent and docetaxel appears to be more toxic than anti-PD1/PD-L1 monoclonal antibodies. Combination of ramucirumab and docetaxel significantly increased toxicity compared to nivolumab alone (OR: 16.27; 95%CI: 10.25–25.85), pembrolizumab alone (OR: 5.38; 95%CI: 3.36–8.62) and atezolizumab (OR: 2.71; 95%CI: 1.95–3.76). In the subsequent setting, pembrolizumab alone appears to be more toxic than nivolumab alone (OR: 3.03; 95%CI: 1.74–5.26).

### Sensitivity analysis

The stepwise sensitivity analysis was performed by omitting studies with high and unclear risk of bias (Supplementary Table S5). Although statistical significance was not remained for some comparisons due to reduce statistical power by omitting studies, the combined hazard ratios maintained similar magnitudes and directions.

## Discussion

Antiangiogenic therapy, immune checkpoint inhibitors, and doublet platinum-based chemotherapy are considered first-line treatments for advanced NSCLC without targeted gene mutations^73,74^. Whether immune checkpoint inhibitors and antiangiogenic therapy are equally effective against advanced NSCLC is unknown. This study conducted an analysis integrating direct and indirect comparisons of both the efficacy and safety of diverse regimens, namely antiangiogenic therapy, immune checkpoint inhibitors, and doublet platinum-based therapy. In this study, pembrolizumab and nivolumab demonstrated potentially more favourable efficacy and tolerability than antiangiogenic therapy and doublet platinum-based therapy; thus, pembrolizumab and nivolumab might be favourable choices for first-line and subsequent treatment, respectively, for NSCLC. However, prospective comparisons of immune checkpoint inhibitors and antiangiogenic therapy in randomised clinical trials are warranted to further address this topic.

The development of immune checkpoint inhibitors has ushered in a new era of cancer treatment. However, given the recent failure of clinical trials of immune checkpoint inhibitors, the role of immune checkpoint inhibitors in cancer treatment requires careful assessment^41,75^. Several ongoing clinical trials are investigating the role of new immune checkpoint inhibitors in cancer treatment and are comparing immune checkpoint inhibitors and antiangiogenic therapy. There are 13 ongoing phase 3 trials comparing immune checkpoint inhibitors and chemotherapy (clinicaltrial.gov ID: NCT02578680; NCT02220894; NCT02576574; NCT02409355; NCT02367794; NCT02367781; NCT02279732; NCT02864251; NCT02775435; NCT02864394; NCT02813785; NCT02613507; and NCT02395172). One ongoing phase 3 trial and one ongoing phase 2 trial are comparing immune checkpoint inhibitors and antiangiogenic therapy in first-line settings (clinicaltrial.gov ID: NCT02366143; NCT02039674). Additionally, the role of a combination of PD-1 inhibitors and CTLA-4 inhibitors in advanced NSCLC treatment are currently being studied in three phase 2 trials and eight phase 3 trials (clinicaltrial.gov ID: NCT03091491; NCT02659059; NCT03057106; NCT03215706; NCT03048136; NCT02477826; NCT02998528; NCT02453282; NCT02542293; NCT02352948; NCT03164616). These studies may provide robust evidence of the role of immune checkpoint inhibitors in advanced NSCLC.

Why PD-1 inhibitors exhibit superior efficacy on OS but not progression-free survival is unclear. Pseudoprogression—that is, existing lesions transiently increasing in size or new lesions temporarily appearing before radiographic improvement occurs—may be a possible cause. Thus, the framework of the Response Evaluation Criteria in Solid Tumors may not be appropriate for detecting the response of immune checkpoint inhibitors^76^. On the other hand, the lack of mortality benefit in antiangiogenic therapy may be related to the resistant mechanism. In the advanced stage and with patients for whom antiangiogenic therapy failed, the tumour may initiate the resistant mechanisms or compensatory pathway to make other drugs ineffective.

The present study utilised the latest data to perform multiple indirect comparisons of immune checkpoint inhibitors, antiangiogenic therapy, and chemotherapy. Nevertheless, this study had several limitations. First, OS, which was the primary endpoint in this study, may be influenced by several factors, such as postprogression treatment and crossover design. In addition, a previous study suggested that OS could not be regarded as the primary endpoint if the postprogression survival time was longer than 12 months^77^. Therefore, we also analysed progression-free survival because it was less affected by postprogression treatment. A second limitation is the potential prognostic role of PD-L1 expression. Given the diverse detecting assays, dynamic expression, and vague cutoff thresholds, conflicting evidence exists regarding whether tumour PD-L1 expression has prognostic value regardless of the use of immune checkpoint inhibitors^55,75,78^. Because most pembrolizumab trials in first-line settings are conducted in patients with high PD-L1 expression, a potential for bias exists when comparing OS between patients with high PD-L1 expression who require first-line pembrolizumab and those treated with doublet platinum-based therapy plus antiangiogenic therapy. Third, the estimated effects of some interventions, particularly first-line treatment with PD-1 inhibitors and subsequent treatment with TKIs, were obtained only from a single trial. Thus, these results must be interpreted with caution. Additionally, adverse event (AE) data were not comprehensively reported in some trials; therefore, we could not extract data on each AE in every trial. Thus, we compared high-grade AEs among the investigated treatments to develop a valuable reference for safety considerations. The various follow-up periods of the included studies were another limitation. However, a subgroup analysis of the follow-up period was not conducted because of the limited data available regarding follow-up periods longer than 2 years. Although it may influence the estimation of efficacy, our findings can still provide information on and a reference for 1-year prognosis of treatment. In addition, due to the limited data, inconsistency analysis could not be conducted. Nevertheless, heterogeneity of the included studies was determined through pairwise and sensitivity analyses to comply with the assumption of indirect comparison^32,33^.

Despite these limitations, our results demonstrated the favourable efficacy and safety of treatment with PD-1 inhibitors (pembrolizumab and nivolumab) relative to antiangiogenic therapy and chemotherapy. These results may have crucial clinical implications for determining which regimen to use in patients with advanced NSCLC without target gene mutations.

In conclusion, based on current evidence, our results revealed that pembrolizumab and nivolumab may be preferable first-line and subsequent treatment options, respectively, for patients with advanced NSCLC without target gene mutations. These findings enhance our understanding of the efficacy and safety of immune checkpoint inhibitors and antiangiogenic therapy in advanced NSCLC.

## Electronic supplementary material

Supplementary Information

## References

[1] Ettinger, D. S. *et al*. Non-small cell lung cancer, version 2.2013. *Journal of the National Comprehensive Cancer Network: JNCCN* **11**, 645–653; quiz653 (2013). 10.6004/jnccn.2013.0084 23744864

[2] Cetin K, Ettinger DS, Hei YJ, O’Malley CD (2011). Survival by histologic subtype in stage IV nonsmall cell lung cancer based on data from the Surveillance, Epidemiology and End Results Program. Clinical epidemiology.

[3] Kwak EL (2010). Anaplastic lymphoma kinase inhibition in non-small-cell lung cancer. The New England journal of medicine.

[4] Lynch TJ (2004). Activating mutations in the epidermal growth factor receptor underlying responsiveness of non-small-cell lung cancer to gefitinib. The New England journal of medicine.

[5] Bazhenova, L. *et al*. An international, multicenter, randomized, double-blind phase III study of maintenance belagenpumatucel-l in non-small cell lung cancer (NSCLC): Updated analysis of patients enrolled within 12 weeks of completion of chemotherapy. *Journal of Clinical Oncology* **32** (2014).

[6] Brahmer, J. R. *et al*. KEYNOTE-024: Phase III trial of pembrolizumab (MK-3475) vs platinum-based chemotherapy as first-line therapy for patients with metastatic non-small cell lung cancer (NSCLC) that expresses programmed cell death ligand 1 (PD-L1). *Journal of Clinical Oncology* **33** (2015).

[7] Carbone DP (2014). A phase III, randomized, open-label trial of nivolumab (anti-PD-1; BMS-936558, ONO-4538) versus investigator’s choice chemotherapy (ICC) as first-line therapy for stage IV or recurrent PD-L1+non-small cell lung cancer (NSCLC). Journal of Clinical Oncology.

[8] Govindan R, Morris JC, Rossi GR, Vahanian NN, Link CJ (2014). NLG-0301: An open-label, randomized phase 2B active control study of second-line tergenpumatucel-L immunotherapy versus docetaxel in patients with progressive or relapsed non-small cell lung cancer (NSCLC). Journal of Clinical Oncology.

[9] Gulley JL (2015). Avelumab (MSB0010718C), an anti-PD-L1 antibody, in advanced NSCLC patients: A phase 1b, open-label expansion trial in patients progressing after platinum-based chemotherapy. Journal of Clinical Oncology.

[10] Mok T (2015). Phase 3 KEYNOTE-042 trial of pembrolizumab (MK-3475) versus platinum doublet chemotherapy in treatment-naive patients (pts) with PD-L1–positive advanced non-small cell lung cancer (NSCLC). Journal of Clinical Oncology.

[11] Nishio M (2015). Phase II studies of nivolumab (anti-PD-1, BMS-936558, ONO-4538) in patients with advanced squamous (sq) or nonsquamous (non-sq) non-small cell lung cancer (NSCLC). Journal of Clinical Oncology.

[12] Planchard D (2015). A phase III study of MEDI4736 (M), an anti-PD-L1 antibody, in monotherapy or in combination with Tremelimumab (T), versus standard of care (SOC) in patients (pts) with advanced non-small cell lung cancer (NSCLC) who have received at least two prior systemic treatment regimens (ARCTIC). Journal of Clinical Oncology.

[13] Reck M (2012). CA184-104: Randomized, multicenter, double-blind, phase III trial comparing the efficacy of ipilimumab (Ipi) with paclitaxel/carboplatin (PC) versus placebo with PC in patients (pts) with stage IV/recurrent non-small cell lung cancer (NSCLC) of squamous histology. Journal of Clinical Oncology.

[14] Callahan MK, Wolchok JD (2013). At the bedside: CTLA-4-and PD-1-blocking antibodies in cancer immunotherapy. Journal of leukocyte biology.

[15] Chen Y, Mu C-Y, Huang J-A (2012). Clinical significance of programmed death-1 ligand-1 expression in patients with non-small cell lung cancer: a 5-year-follow-up study. Tumori.

[16] Pardoll DM (2012). The blockade of immune checkpoints in cancer immunotherapy. Nature reviews. Cancer.

[17] Velcheti V (2014). Programmed death ligand-1 expression in non-small cell lung cancer. Laboratory investigation.

[18] Tumeh PC (2014). PD-1 blockade induces responses by inhibiting adaptive immune resistance. Nature.

[19] Barlesi, F. *et al*. Primary analysis from OAK, a randomized phase III study comparing atezolizumab with docetaxel in 2L/3L NSCLC. *Annals of Oncology* **27** (2016).

[20] Borghaei H (2015). Nivolumab versus docetaxel in advanced nonsquamous non–small-cell lung cancer. New England Journal of Medicine.

[21] Brahmer J (2015). Nivolumab versus docetaxel in advanced squamous-cell non–small-cell lung cancer. New England Journal of Medicine.

[22] Fehrenbacher L (2016). Atezolizumab versus docetaxel for patients with previously treated non-small-cell lung cancer (POPLAR): a multicentre, open-label, phase 2 randomised controlled trial. The Lancet.

[23] De Bock K, Mazzone M, Carmeliet P (2011). Antiangiogenic therapy, hypoxia, and metastasis: risky liaisons, or not?. Nature reviews. Clinical oncology.

[24] Garon EB (2014). Ramucirumab plus docetaxel versus placebo plus docetaxel for second-line treatment of stage IV non-small-cell lung cancer after disease progression on platinum-based therapy (REVEL): a multicentre, double-blind, randomised phase 3 trial. The Lancet.

[25] Sandler A (2007). Bevacizumab in non small cell lung cancer. Clinical cancer research: an official journal of the American Association for Cancer Research.

[26] Sandler A (2006). Paclitaxel–carboplatin alone or with bevacizumab for non–small-cell lung cancer. New England Journal of Medicine.

[27] Carmeliet P (2005). Angiogenesis in life, disease and medicine. Nature.

[28] Folkman J (2007). Angiogenesis: an organizing principle for drug discovery?. Nature reviews. Drug discovery.

[29] Kerbel RS (2008). Tumor angiogenesis. The New England journal of medicine.

[30] Raphael J (2017). Antiangiogenic Therapy in Advanced Non-small-cell Lung Cancer: A Meta-analysis of Phase III Randomized Trials. Clinical lung cancer.

[31] Zhuansun Y (2017). Anti-PD-1/PD-L1 antibody versus conventional chemotherapy for previously-treated, advanced non-small-cell lung cancer: a meta-analysis of randomized controlled trials. Journal of thoracic disease.

[32] Kiefer C, Sturtz S, Bender R (2015). Indirect Comparisons and Network Meta-Analyses. Deutsches Arzteblatt international.

[33] Kim H, Gurrin L, Ademi Z, Liew D (2014). Overview of methods for comparing the efficacies of drugs in the absence of head-to-head clinical trial data. British journal of clinical pharmacology.

[34] Salanti G (2012). Indirect and mixed-treatment comparison, network, or multiple-treatments meta-analysis: many names, many benefits, many concerns for the next generation evidence synthesis tool. Research synthesis methods.

[35] Donegan S, Williamson P, D’Alessandro U, Tudur Smith C (2013). Assessing key assumptions of network meta-analysis: a review of methods. Research synthesis methods.

[36] White IR, Barrett JK, Jackson D, Higgins JP (2012). Consistency and inconsistency in network meta-analysis: model estimation using multivariate meta-regression. Research synthesis methods.

[37] Tonin FS, Rotta I, Mendes AM, Pontarolo R (2017). Network meta-analysis: a technique to gather evidence from direct and indirect comparisons. Pharmacy practice.

[38] Harbord RM, Higgins J (2008). Meta-regression in Stata. Meta.

[39] Higgins, J. P. T. G., S (editors) (The Cochrane Collaboration, 2011).

[40] Belani CP (2014). Randomized phase II study of pemetrexed/cisplatin with or without axitinib for non-squamous non-small-cell lung cancer. BMC cancer.

[41] Carbone DP (2017). First-Line Nivolumab in Stage IV or Recurrent Non-Small-Cell Lung Cancer. The New England journal of medicine.

[42] Doebele RC (2015). Phase 2, randomized, open-label study of ramucirumab in combination with first-line pemetrexed and platinum chemotherapy in patients with nonsquamous, advanced/metastatic non-small cell lung cancer. Cancer.

[43] Heymach JV (2008). Randomized phase II study of vandetanib alone or with paclitaxel and carboplatin as first-line treatment for advanced non-small-cell lung cancer. Journal of clinical oncology: official journal of the American Society of Clinical Oncology.

[44] Johnson DH (2004). Randomized phase II trial comparing bevacizumab plus carboplatin and paclitaxel with carboplatin and paclitaxel alone in previously untreated locally advanced or metastatic non-small-cell lung cancer. Journal of clinical oncology: official journal of the American Society of Clinical Oncology.

[45] Langer CJ (2016). Carboplatin and pemetrexed with or without pembrolizumab for advanced, non-squamous non-small-cell lung cancer: a randomised, phase 2 cohort of the open-label KEYNOTE-021 study. The Lancet. Oncology.

[46] Lynch TJ (2012). Ipilimumab in combination with paclitaxel and carboplatin as first-line treatment in stage IIIB/IV non-small-cell lung cancer: results from a randomized, double-blind, multicenter phase II study. Journal of clinical oncology: official journal of the American Society of Clinical Oncology.

[47] Niho S (2012). Randomized phase II study of first-line carboplatin-paclitaxel with or without bevacizumab in Japanese patients with advanced non-squamous non-small-cell lung cancer. Lung cancer (Amsterdam, Netherlands).

[48] Paz-Ares LG (2012). Phase III, randomized, double-blind, placebo-controlled trial of gemcitabine/cisplatin alone or with sorafenib for the first-line treatment of advanced, nonsquamous non-small-cell lung cancer. Journal of clinical oncology: official journal of the American Society of Clinical Oncology.

[49] Reck M (2016). Pembrolizumab versus Chemotherapy for PD-L1-Positive Non-Small-Cell Lung Cancer. The New England journal of medicine.

[50] Reck M (2009). Phase III trial of cisplatin plus gemcitabine with either placebo or bevacizumab as first-line therapy for nonsquamous non-small-cell lung cancer: AVAil. Journal of clinical oncology: official journal of the American Society of Clinical Oncology.

[51] Scagliotti G (2010). Phase III study of carboplatin and paclitaxel alone or with sorafenib in advanced non-small-cell lung cancer. Journal of clinical oncology: official journal of the American Society of Clinical Oncology.

[52] Wang Y (2011). Randomize trial of cisplatin plus gemcitabine with either sorafenib or placebo as first-line therapy for non-small cell lung cancer. Zhongguo fei ai za zhi=Chinese journal of lung cancer.

[53] Zhou C (2015). BEYOND: A Randomized, Double-Blind, Placebo-Controlled, Multicenter, Phase III Study of First-Line Carboplatin/Paclitaxel Plus Bevacizumab or Placebo in Chinese Patients With Advanced or Recurrent Nonsquamous Non-Small-Cell Lung Cancer. Journal of clinical oncology: official journal of the American Society of Clinical Oncology.

[54] Zukin M (2013). Randomized phase III trial of single-agent pemetrexed versus carboplatin and pemetrexed in patients with advanced non-small-cell lung cancer and Eastern Cooperative Oncology Group performance status of 2. Journal of clinical oncology: official journal of the American Society of Clinical Oncology.

[55] Gainor JF (2017). Programmed death-ligand 1 testing in patients with non-small cell lung cancer: Ready for prime time?. Cancer.

[56] Rogerio Lilenbaum, M. Z. *et al*. Isabele AvilaSmall, Carlos G. M. Ferreira. in *2012 ASCO Annual Meeting* Vol. 30 (2012).

[57] Belvedere O (2011). A randomised phase II study of docetaxel/oxaliplatin and docetaxel in patients with previously treated non-small cell lung cancer: an Alpe-Adria Thoracic Oncology Multidisciplinary group trial (ATOM 019). European journal of cancer (Oxford, England: 1990).

[58] Brahmer J (2015). Nivolumab versus Docetaxel in Advanced Squamous-Cell Non-Small-Cell Lung Cancer. The New England journal of medicine.

[59] Hanna N (2004). Randomized phase III trial of pemetrexed versus docetaxel in patients with non-small-cell lung cancer previously treated with chemotherapy. Journal of clinical oncology: official journal of the American Society of Clinical Oncology.

[60] Herbst RS (2016). Pembrolizumab versus docetaxel for previously treated, PD-L1-positive, advanced non-small-cell lung cancer (KEYNOTE-010): a randomised controlled trial. Lancet (London, England).

[61] Herbst RS (2010). Vandetanib plus docetaxel versus docetaxel as second-line treatment for patients with advanced non-small-cell lung cancer (ZODIAC): a double-blind, randomised, phase 3 trial. The Lancet. Oncology.

[62] Heymach JV (2007). Randomized, placebo-controlled phase II study of vandetanib plus docetaxel in previously treated non small-cell lung cancer. Journal of clinical oncology: official journal of the American Society of Clinical Oncology.

[63] Pallis AG (2010). A randomized phase III study of the docetaxel/carboplatin combination versus docetaxel single-agent as second line treatment for patients with advanced/metastatic non-small cell lung cancer. BMC cancer.

[64] Ramlau R (2012). Aflibercept and Docetaxel versus Docetaxel alone after platinum failure in patients with advanced or metastatic non-small-cell lung cancer: a randomized, controlled phase III trial. Journal of clinical oncology: official journal of the American Society of Clinical Oncology.

[65] Reck M (2014). Docetaxel plus nintedanib versus docetaxel plus placebo in patients with previously treated non-small-cell lung cancer (LUME-Lung 1): a phase 3, double-blind, randomised controlled trial. The Lancet. Oncology.

[66] Rittmeyer A (2017). Atezolizumab versus docetaxel in patients with previously treated non-small-cell lung cancer (OAK): a phase 3, open-label, multicentre randomised controlled trial. Lancet (London, England).

[67] Sun Y (2013). Second-line pemetrexed versus docetaxel in Chinese patients with locally advanced or metastatic non-small cell lung cancer: a randomized, open-label study. Lung cancer (Amsterdam, Netherlands).

[68] Yoh K (2016). A randomized, double-blind, phase II study of ramucirumab plus docetaxel vs placebo plus docetaxel in Japanese patients with stage IV non-small cell lung cancer after disease progression on platinum-based therapy. Lung cancer (Amsterdam, Netherlands).

[69] Ardizzoni A (2012). Pemetrexed versus pemetrexed and carboplatin as second-line chemotherapy in advanced non-small-cell lung cancer: results of the GOIRC 02-2006 randomized phase II study and pooled analysis with the NVALT7 trial. Journal of clinical oncology: official journal of the American Society of Clinical Oncology.

[70] Smit EF (2009). Randomized phase II and pharmacogenetic study of pemetrexed compared with pemetrexed plus carboplatin in pretreated patients with advanced non-small-cell lung cancer. Journal of clinical oncology: official journal of the American Society of Clinical Oncology.

[71] Hanna NH (2016). Nintedanib plus pemetrexed versus placebo plus pemetrexed in patients with relapsed or refractory, advanced non-small cell lung cancer (LUME-Lung 2): A randomized, double-blind, phase III trial. Lung cancer (Amsterdam, Netherlands).

[72] Heist RS (2014). CALGB 30704 (Alliance): A randomized phase II study to assess the efficacy of pemetrexed or sunitinib or pemetrexed plus sunitinib in the second-line treatment of advanced non-small-cell lung cancer. Journal of thoracic oncology: official publication of the International Association for the Study of Lung Cancer.

[73] Ettinger DS (2017). Non-Small Cell Lung Cancer, Version 5.2017, NCCN Clinical Practice Guidelines in Oncology. Journal of the National Comprehensive Cancer Network: JNCCN.

[74] Hanna, N. *et al*. Systemic Therapy for Stage IV Non-Small-Cell Lung Cancer: American Society of Clinical Oncology Clinical Practice Guideline Update. *Journal of clinical oncology: official journal of the American Society of Clinical Oncology*, Jco2017746065, 10.1200/jco.2017.74.6065 (2017).

[75] Peters S, Kerr KM, Stahel R (2018). PD-1 blockade in advanced NSCLC: A focus on pembrolizumab. Cancer treatment reviews.

[76] Chiou VL, Burotto M (2015). Pseudoprogression and Immune-Related Response in Solid Tumors. Journal of clinical oncology: official journal of the American Society of Clinical Oncology.

[77] Broglio KR, Berry DA (2009). Detecting an overall survival benefit that is derived from progression-free survival. Journal of the National Cancer Institute.

[78] Valecha GK (2017). Anti-PD-1/PD-L1 antibodies in non-small cell lung cancer: the era of immunotherapy. Expert review of anticancer therapy.

