# Supplementary material for: Indirect comparison of efficacy and safety between immune checkpoint inhibitors and antiangiogenic therapy in advanced non–small-cell lung cancer
Source: Sci Rep. 2018 Jun 26;8:9686. doi: 10.1038/s41598-018-27994-x (PMC6018789; doi:10.1038/s41598-018-27994-x)
Supplement: Supplementary file 1 — Supplementary Information [file 41598_2018_27994_MOESM1_ESM.docx]

**Indirect comparison of efficacy and safety between immune checkpoint inhibitors and antiangiogenic therapy in advanced non–small-cell lung cancer**

Jin-Hua Chen ^1, 2#^, Jia-Lian Yang^3#^, Che-Yi Chou^4^, Jiun-Yi Wang^5^, Chin-Chuan Hung^3, 6^*

^1^ Graduate Institute of Data Science, College of Management, Taipei Medical University; ^2^ Research Center of Biostatistics, Taipei Medical University; ^3^ Department of Pharmacy, College of Pharmacy, China Medical University, 91 Hsueh-Shih Road, Taichung 40402, Taiwan, R.O.C.;^4^ Kidney Institute and Division of Nephrology, Department of Internal Medicine, China Medical University Hospital, 2 Yude Road, Taichung 40447, Taiwan, R.O.C.; ^5^ Department of Healthcare Administration, Asia University, Wufeng, Taichung 41354, Taiwan, R.O.C.; ^6^ Department of Pharmacy, China Medical University Hospital, 2 Yude Road, Taichung 40447, Taiwan, R.O.C.

^#^ These authors contributed equally.

* Corresponding Author

E-mail: [cc0206hung@gmail.com](mailto:cc0206hung@gmail.com) (C-CH)

**Supplementary table S1. PRISMA NMA Checklist of Items to Include When Reporting A Systematic Review Involving a Network Meta-analysis**

| **Section/Topic** | **Item #** | **Checklist Item** | **Reported on Page #** |
| --- | --- | --- | --- |
| **TITLE** |  |  |  |
| Title | 1 | Identify the report as a systematic review *incorporating a network meta-analysis (or related form of meta-analysis).* | ***1*** |
| **ABSTRACT** |  |  |  |
| Structured summary | 2 | Provide a structured summary including, as applicable:  **Background:** main objectives  **Methods:** data sources; study eligibility criteria, participants, and interventions; study appraisal; and *synthesis methods, such as network meta-analysis.*  **Results:** number of studies and participants identified; summary estimates with corresponding confidence/credible intervals; *treatment rankings may also be discussed. Authors may choose to summarize pairwise comparisons against a chosen treatment included in their analyses for brevity.*  **Discussion/Conclusions:** limitations; conclusions and implications of findings.  **Other:** primary source of funding; systematic review registration number with registry name. | 2-3 |
| **INTRODUCTION** |  |  |  |
| Rationale | 3 | Describe the rationale for the review in the context of what is already known*, including mention of why a network meta-analysis has been conducted.* | ***4-5*** |
| Objectives | 4 | Provide an explicit statement of questions being addressed, with reference to participants, interventions, comparisons, outcomes, and study design (PICOS). | 6 |
| **METHODS** |  |  |  |
| Protocol and registration | 5 | Indicate whether a review protocol exists and if and where it can be accessed (e.g., Web address); and, if available, provide registration information, including registration number. | 6 |
| Eligibility criteria | 6 | Specify study characteristics (e.g., PICOS, length of follow-up) and report characteristics (e.g., years considered, language, publication status) used as criteria for eligibility, giving rationale. *Clearly describe eligible treatments included in the treatment network, and note whether any have been clustered or merged into the same node (with justification).* | ***6-7*** |
| Information sources | 7 | Describe all information sources (e.g., databases with dates of coverage, contact with study authors to identify additional studies) in the search and date last searched. | 6 |
| Search | 8 | Present full electronic search strategy for at least one database, including any limits used, such that it could be repeated. | 6 |
| Study selection | 9 | State the process for selecting studies (i.e., screening, eligibility, included in systematic review, and, if applicable, included in the meta-analysis). | 6-7 |
| Data collection process | 10 | Describe method of data extraction from reports (e.g., piloted forms, independently, in duplicate) and any processes for obtaining and confirming data from investigators. | 7 |
| Data items | 11 | List and define all variables for which data were sought (e.g., PICOS, funding sources) and any assumptions and simplifications made. | 7-8 |
| **Geometry of the network** | **S1** | Describe methods used to explore the geometry of the treatment network under study and potential biases related to it. This should include how the evidence base has been graphically summarized for presentation, and what characteristics were compiled and used to describe the evidence base to readers. | ***8-10*** |
| Risk of bias within individual studies | 12 | Describe methods used for assessing risk of bias of individual studies (including specification of whether this was done at the study or outcome level), and how this information is to be used in any data synthesis. | 7 |
| Summary measures | 13 | State the principal summary measures (e.g., risk ratio, difference in means). *Also describe the use of additional summary measures assessed, such as treatment rankings and surface under the cumulative ranking curve (SUCRA) values, as well as modified approaches used to present summary findings from meta-analyses.* | 8-9 |
| Planned methods of analysis | 14 | Describe the methods of handling data and combining results of studies for each network meta-analysis. This should include, but not be limited to:   - *Handling of multi-arm trials;* - *Selection of variance structure;* - *Selection of prior distributions in Bayesian analyses; and* - *Assessment of model fit.* | 8-9 |
| **Assessment of Inconsistency** | **S2** | Describe the statistical methods used to evaluate the agreement of direct and indirect evidence in the treatment network(s) studied. Describe efforts taken to address its presence when found. | 9 |
| Risk of bias across studies | 15 | Specify any assessment of risk of bias that may affect the cumulative evidence (e.g., publication bias, selective reporting within studies). | **7** |
| Additional analyses | 16 | Describe methods of additional analyses if done, indicating which were pre-specified. This may include, but not be limited to, the following:   - Sensitivity or subgroup analyses; - Meta-regression analyses; - *Alternative formulations of the treatment network; and* - *Use of alternative prior distributions for Bayesian analyses (if applicable).* | ***9*** |
| **RESULTS†** |  |  |  |
| Study selection | 17 | Give numbers of studies screened, assessed for eligibility, and included in the review, with reasons for exclusions at each stage, ideally with a flow diagram. | 11  Figure 1 |
| **Presentation of network structure** | **S3** | Provide a network graph of the included studies to enable visualization of the geometry of the treatment network. | *supplementary Figure* ***S1*** |
| **Summary of network geometry** | **S4** | Provide a brief overview of characteristics of the treatment network. This may include commentary on the abundance of trials and randomized patients for the different interventions and pairwise comparisons in the network, gaps of evidence in the treatment network, and potential biases reflected by the network structure. | ***10*** |
| Study characteristics | 18 | For each study, present characteristics for which data were extracted (e.g., study size, PICOS, follow-up period) and provide the citations. | supplementary table S2 |
| Risk of bias within studies | 19 | Present data on risk of bias of each study and, if available, any outcome level assessment. | 11  ***Supplementary Figure S2*** |
| Results of individual studies | 20 | For all outcomes considered (benefits or harms), present, for each study: 1) simple summary data for each intervention group, and 2) effect estimates and confidence intervals. *Modified approaches may be needed to deal with information from larger networks.* | ***Supplementary***  ***Figure S3-S8, S10-S12*** |
| Synthesis of results | 21 | Present results of each meta-analysis done, including confidence/credible intervals. *In larger networks, authors may focus on comparisons versus a particular comparator (e.g. placebo or standard care), with full findings presented in an appendix. League tables and forest plots may be considered to summarize pairwise comparisons.* If additional summary measures were explored (such as treatment rankings), these should also be presented. | ***Table 1 , 2***  ***Supplementary***  ***Table S3-S4***  supplementary Figure S***9, Figure 2, Figure 3*** |
| **Exploration for inconsistency** | **S5** | Describe results from investigations of inconsistency. This may include such information as measures of model fit to compare consistency and inconsistency models, *P* values from statistical tests, or summary of inconsistency estimates from different parts of the treatment network. | ***NA*** |
| Risk of bias across studies | 22 | Present results of any assessment of risk of bias across studies for the evidence base being studied. | ***Supplementary Figure S2*** |
| Results of additional analyses | 23 | Give results of additional analyses, if done (e.g., sensitivity or subgroup analyses, meta-regression analyses*, alternative network geometries studied, alternative choice of prior distributions for Bayesian analyses,* and so forth). | ***Supplementary***  ***Table S5*** |
| **DISCUSSION** |  |  |  |
| Summary of evidence | 24 | Summarize the main findings, including the strength of evidence for each main outcome; consider their relevance to key groups (e.g., healthcare providers, users, and policy-makers). | P18 |
| Limitations | 25 | Discuss limitations at study and outcome level (e.g., risk of bias), and at review level (e.g., incomplete retrieval of identified research, reporting bias). *Comment on the validity of the assumptions, such as transitivity and consistency. Comment on any concerns regarding network geometry (e.g., avoidance of certain comparisons).* | P20 |
| Conclusions | 26 | Provide a general interpretation of the results in the context of other evidence, and implications for future research. | P20 |
| **FUNDING** |  |  |  |
| Funding | 27 | Describe sources of funding for the systematic review and other support (e.g., supply of data); role of funders for the systematic review. This should also include information regarding whether funding has been received from manufacturers of treatments in the network and/or whether some of the authors are content experts with professional conflicts of interest that could affect use of treatments in the network. | ***NA*** |

# Supplementary table S2. Characteristics of randomized controlled trials included in the indirect comparison analysis.

| **Study** | **Type** | **Phase** | **Allocation** | **sample size** | **intervention** | **dose** | **Follow-up** |
| --- | --- | --- | --- | --- | --- | --- | --- |
| **First-line therapy** | | | | | | | |
| 2015 Zhou, C. | Advanced NSCLC | 3 | D | 138 | Carboplatin/paclitaxel + bevacizumab | Bevacizumab: IV At 15 mg/kg , on day 1 of each cycle  Carboplatin AUC 6 mg/ml · min, IV and paclitaxel (175 mg/ m²) IV on day 1 of each 3-week cycle x 6 cycles | 36 months  Median : 28.1 months |
|  |  |  |  | 138 | Carboplatin/paclitaxel | Carboplatin :AUC 6 mg/ml · min , IV and paclitaxel (175 mg/ m²) IV on day 1 of each 3-week cycle x 6 cycles | 36 months  Median : 26.9 months |
| 2012Niho, S. | Advanced NSCLC | 2 | O | 121 | Carboplatin-paclitaxel + bevacizumab | Carboplatin: AUC of 6 mg/(ml min)  Paclitaxel: 200 mg/ m²  Bevacizumab : 15 mg/kg. | 36 months |
|  |  |  |  | 59 | Carboplatin-paclitaxel | Carboplatin : AUC of 6 mg/(ml min) paclitaxel : 200 mg/ m² | 36 months |
| 2010Reck, M. | Advanced NSCLC | 3 | D | 351 | Cisplatin + gemcitabine + bevacizumab | Bevacizumab 15 mg/kg Cisplatin : IV at 80 mg/ m² on day 1  Gemcitabine : IV at 1250 mg/ m² on days 1 and 8. | 32 months (Median :12.5 months) |
|  |  |  |  | 347 | Cisplatin + gemcitabine | Cisplatin : IV. At 80 mg/ m² on day 1  Gemcitabine : IV at 1250 mg/ m² on days 1 and 8. | 32 months (Median :12.5 months) |
| 2006Sandler, A. | Advanced NSCLC | 3 | D | 444 | Paclitaxel + carboplatin+ bevacizumab | Paclitaxel: 200 mg per square meter of body-surface area  carboplatin : AUC 6.0 mg/ml · min bevacizumab 15 mg /kg | 42 months  Median: 19 months |
|  |  |  |  | 434 | Paclitaxel + carboplatin | Paclitaxel: 200 mg per square meter of body-surface area  carboplatin : AUC 6.0 mg mg/ml · min | 42 months  Median: 19 months |

**D: double-blinded; O: open-labeled**

**Supplementary table S2. (continued)**

| **Study** | **Type** | **Phase** | **Allocation** | **Sample size** | **intervention** | **dose** | **Follow-up** |
| --- | --- | --- | --- | --- | --- | --- | --- |
| 2004Johnson, D. H. | Advanced NSCLC | 2 |  | 35 | Bevacizumab + carboplatin + paclitaxel | Bevacizumab 15 mg/kg  Paclitaxel: IV 200 mg/ m² every 3 weeks.  Carboplatin : IV AUC 6 mg/ml · min | 60 months |
|  |  |  |  | 32 | Carboplatin and paclitaxel | Paclitaxel: IV 200 mg/ m² was every 3 weeks.  Carboplatin : IV AUC 6 mg/ml · min | 60 months |
| 2012Paz-Ares, L. G. | Advanced NSCLC | 3 | D | 452 | Cisplatin + gemcitabine + sorafenib | Sorafenib (400 mg twice a day)  Gemcitabine (1,250 mg/ m² per day)  Cisplatin (75 mg/ m²) | 33.3 months |
|  |  |  |  | 452 | Cisplatin + gemcitabine | Gemcitabine (1,250 mg/ m² per day)  Cisplatin (75 mg/ m²) | 33.3 months |
| 2011Wang, Y. | Advanced NSCLC |  |  | 18 | Cisplatin + gemcitabine+ Sorafenib | Gemcitabine: 1250 mg/ m²  Cisplatin: 75 mg/ m²  Sorafenib : 400 mg BID | 50 months |
|  |  |  |  | 12 | Cisplatin + gemcitabine | Gemcitabine: 1250 mg/ m²  Cisplatin : 75 mg/ m² | 50 months |
| 2010Scagliotti, G. | Advanced NSCLC | 3 | D | 464 | Carboplatin + paclitaxel + sorafenib | Paclitaxel: (200 mg/ m² IV)  Carboplatin: (AUC 6 mg/ml ·min IV)  Sorafenib : (400 mg orally twice a day) | 20 months |
|  |  |  |  | 462 | Carboplatin + paclitaxel | Paclitaxel: (200 mg/ m² IV)  Carboplatin: (AUC 6 mg/ml · min IV) | 20 months |

**D: double-blinded; O: open-labeled**

**Supplementary table S2. (continued)**

| **Study** | **Type** | **Phase** | **Allocation** | **Sample size** | **intervention** | **dose** | **Follow-up** |  |
| --- | --- | --- | --- | --- | --- | --- | --- | --- |
| 2008Heymach, J. V. | Advanced NSCLC | 2 | partially blinded | 56 | Paclitaxel + carboplatin+ Vandetanib | paclitaxel (200 mg/ m²). carboplatin (AUC 6 mg/mL/min) Vandetanib 300 mg arm | 30 months  Median: 21.4 months | |
|  |  |  |  | 52 | Paclitaxel + carboplatin | paclitaxel (200 mg/ m²). carboplatin (AUC 6 mg/mL/min) | 30 months  Median:19.2 months | |
| 2015Doebele RC | Advanced NSCLC | 2 | O | 71 | Ramucirumab + Pemetrexed + Carboplatin/Cisplatin | Pemetrexed: 500 mg/m² on Day 1 of every 21-day cycle.  Carboplatin (AUC 6 mg/ml · min): Day 1 of every 21-day cycle.  Cisplatin: 75 mg/m² IV on Day 1 of every 21-day cycle. | 30 months | |
|  |  |  |  | 69 | Pemetrexed +  Carboplatin/ Cisplatin | Ramucirumab: 10 mg/kg Day 1 of every 21-day cycle.  Pemetrexed: 500 mg/m² on Day 1 of every 21-day cycle.  Carboplatin (AUC 6 mg/ml · min): Day 1 of every 21-day cycle.  Cisplatin: 75 mg/m² IV on Day 1 of every 21-day cycle. | 30 months | |
| 2014  Belani CP | Advanced NSCLC | 2 | O | 58 | Axitinib + Pemetrexed/Cisplatin | Axitinib 5mg BID po up to max 10mg BID po paused for 3 days before each cycle of concomitant chemotherapy  Pemetrexed(500mg/ m²)/Cisplatin(75mg/ m²) x max 6 cycles | 36 months | |
|  |  |  |  | 57 | Pemetrexed/Cisplatin | Pemetrexed(500mg/ m²)/Cisplatin(75mg/ m²) x max 6 cycles | 36 months | |
| 2013Zukin, M | Advanced NSCLC | 3 | O | 102 | pemetrexed | pemetrexed 500 mg/ m² Course: on day 1 every 21 days for up to four cycles. | 36 months  Medina : 27.5 months (95% CI, 20.5 to 34.5) | |
|  |  |  |  | 103 | carboplatin + pemetrexed | carboplatin: AUC 5 mg/ml · min  pemetrexed: 500 mg/ m², on day 1 every 21 days for up to 4 cycles. | 36 months  Median :27.5 months (95% CI, 20.5 to 34.5) | |

**D: double-blinded; O: open-labeled**

**Supplementary table S2. (continued)**

| **Study** | **Type** | **Phase** | **Allocation** | **Sample size** | **intervention** | **dose** | **Follow-up** |
| --- | --- | --- | --- | --- | --- | --- | --- |
| 2012Rogerio L. (Abstract) | Advanced NSCLC | 3 |  | 102 | pemetrexed | pemetrexed :500 mg/ m² , every 3 weeks for 4 cycles | NA |
|  |  |  |  | 103 | carboplatin + pemetrexed | carboplatin : AUC 5 mg/ml · min. pemetrexed: 500 mg/ m²  every 3 weeks for 4 cycles | NA |
| 2012Lynch TJ | Advanced NSCLC | 2 | D | 70 | ipilimumab + paclitaxel + carboplatin | Ipilimumab (10 mg/kg)  paclitaxel (175 mg/ m²) , carboplatin (AUC, 6 mg/ml · min) | 26 months |
|  |  |  |  | 66 | carboplatin + paclitaxel | paclitaxel (175 mg/ m²) , carboplatin (AUC, 8 mg/ml · min) | 26 months |
| NCT01285609 | Advanced NSCLC | 3 | D | 479 | Ipilimumab + Paclitaxel/Carboplatin | Ipilimumab: IV, 10 mg/kg.Once every 3 weeks for 4 doses and then every 12 weeks.  Paclitaxel: IV, 175 mg/m². Once every 3 weeks for 6 doses Carboplatin: IV, AUC 6 mg/ml · min. Once every 3 weeks for 6 doses | 48 months |
|  |  |  |  | 477 | Placebo + Paclitaxel/Carboplatin | Paclitaxel: IV, 175 mg/m². Once every 3 weeks for 6 doses Carboplatin: IV, AUC 6 mg/ml · min. Once every 3 weeks for 6 doses | 48 months |
| 2016Reck M. | Advanced NSCLC  PD-L1(+) | 3 | O | 154 | Pembrolizumab | fixed dose of 200 mg every 3 weeks | 36 months  Median: 11.2 months |
|  |  |  |  | 151 | Doublet Platinum-based chemotherapy | Investigator dose | 36 months  Median: 11.2 months |

**D: double-blinded; O: open-labeled**

**Supplementary table S2. (continued)**

| **Study** | **Type** | **Phase** | **Allocation** | **Sample size** | **intervention** | **dose** | **Follow-up** |
| --- | --- | --- | --- | --- | --- | --- | --- |
| 2016Langer CJ | Advanced NSCLC | 2 | O | 60 | Pembrolizumab +  Carboplatin+ pemetrexed | 4 cycles of pembrolizumab 200 mg  pemetrexed 500 mg/m²  carboplatin AUC 5 mg/ml · min every 3 weeks | 20 months  Median :10·6 months (IQR 8·2-13·3). |
|  |  |  |  | 63 | Carboplatin+ pemetrexed | pemetrexed 500 mg/m²  carboplatin AUC 5 mg/ml · min every 3 weeks | 20 months  Median :10·6 months (IQR 8·2–13·3). |
| 2017 D.P. Carbone | Advanced NSCLC  PD-L1(+) | 3 | O | 267 | Nivolumab | 3 mg/kg of body weight every 2 weeks | 27 months  minimum: 13.7 months |
|  |  |  |  | 263 | Doublet Platinum-based chemotherapy | Investigator dose every 3 weeks for four to six cycles | 27 months  minimum: 13.7 months |
| **Subsequent therapy** | | | | | | | |
| 2010Herbst, R. S. | Advanced NSCLC | 3 | D | 694 | Vandetanib + docetaxel | Vandetanib (100 mg/day)  docetaxel (75 mg/m² IV every 21 days; maximum 6 cycles) | 27 months ; Median : 12·8 months |
|  |  |  |  | 697 | docetaxel | docetaxel (75 mg/m² IV every 21 days; maximum 6 cycles) | 27 months ; Median :12·9 months |
| 2007Heymach, J. V. | Advanced NSCLC | 2 | D | 42 | Vandetanib+ docetaxel | Docetaxel 75 mg/ m² + Vandetanib 100 mg | 34 months |
|  |  |  |  | 41 | docetaxel | Docetaxel 75 mg/ m² | 34 months |
| 2012Ramlau R | Advanced NSCLC | 3 | D | 457 | Placebo/Docetaxel | Docetaxel 75 mg/m² in 250 mL on Day 1 every 3 weeks. | 36 months |
|  |  |  |  | 456 | Aflibercept/Docetaxel | Aflibercept 6 mg/kg on Day 1, every 3 weeks. Docetaxel 75 mg/m² in 250 mL on Day 1 every 3 weeks. | 36 months |
| 2016 Kiyotaka | Advanced NSCLC | 2 | D | 76 | Ramucirumab + Docetaxel | Ramucirumab : 10 (mg/kg) on Day 1 of every 21 day cycle, Docetaxel: 60 (mg/ m²) on Day 1 of every 21 day cycle, | 30months |
|  |  |  |  | 81 | Docetaxel | Docetaxel: 60 (mg/ m²) on Day 1 of every 21 day cycle, | 30 months |

**D: double-blinded; O: open-labeled**

**Supplementary table S2. (continued)**

| **Study** | **Type** | **Phase** | **Allocation** | **Sample size** | **intervention** | **dose** | **Follow-up** |
| --- | --- | --- | --- | --- | --- | --- | --- |
| 2014Reck, M. | Advanced NSCLC | 3 | D | 655 | docetaxel + nintedanib | docetaxel 75 mg/m² by IV nintedanib 200 mg twice daily orally | 36 months  Median: PFS7·1 months (3·8–11·0)  Median: OS 31·7 months (27·8–36·2) |
|  |  |  |  | 659 | docetaxel | docetaxel 75 mg/m² by IV | 36 months  Median: PFS7·1 months (3·8–11·0)  Median: OS 31·7 months (27·8–36·2) |
| 2014Garon, E. B. | Advanced NSCLC | 3 | D | 628 | Ramucirumab + Docetaxel | IV docetaxel 75 mg/m²  IV ramucirumab 10 mg/kg | Median : 9·5 months [IQR 4·4–14·9] |
|  |  |  |  | 625 | Docetaxel | IV docetaxel 75 mg/m² | Median ·8·8 months [3·7–13·7] |
| 2016Fehrenbacher, L. | Advanced NSCLC | 2 | O | 144 | Atezolizumab | 1200 mg ﬁxed dose | 20 months  Median :14·8 months (range 0·2- 19·6) |
|  |  |  |  | 143 | Docetaxel | 75 mg/m² | 20 months  Median : 15·7 months (range 0·1–18·7) |
| 2015Borghaei H | Advanced NSCLC | 3 | O | 292 | Nivolumab | nivolumab : 3 mg/kg | 13.2-27 months |
|  |  |  |  | 290 | Docetaxel | docetaxel at : 75 mg/ m² of BSA every 3 weeks | 13.3-27 months |
| 2015Brahmer J | Advanced NSCLC | 3 | O | 135 | Nivolumab | nivolumab : 3 mg/kg | 11-24 months |
|  |  |  |  | 137 | Docetaxel | docetaxel : 75 mg/ m² | 12 -24months |

**D: double-blinded; O: open-labeled**

**Supplementary table S2. (continued)**

| **Study** | **Type** | **Phase** | **Allocation** | **Sample size** | **intervention** | **dose** | **Follow-up** |
| --- | --- | --- | --- | --- | --- | --- | --- |
| 2015Herbst, R. S. | PD-L1(+)  Advanced NSCLC | 2/3 | O | 344 | Pembrolizumab 2 mg/kg | pembrolizumab 2 mg/kg every 3 weeks | 25 months  Median :13·1months(IQR 8·6–17·7) |
|  |  |  |  | 343 | docetaxel 75 mg/m² | docetaxel 75 mg/m² every 3 weeks | 25 months  Median :13·1 months (IQR 8·6–17·7) |
| Achim 2017 | Advanced NSCLC | 3 | O | 425 | Atezolizumab | Atezolizumab : iv, 1200 mg every 3 weeks | 27 months  Median : 21 months |
|  |  |  |  | 425 | Docetaxel | Docetaxel :75 mg/m² every 3 weeks | 27 months  Median : 21 months |
| 2004Hanna, N. | Advanced NSCLC | 3 | O | 283 | Pemetrexed | pemetrexed: 500 mg/ m² ; on day 1 every 21 days for up to 4 cycles. | 20 months ;Median : 7.5 months |
|  |  |  |  | 288 | Docetaxel | docetaxel 75 mg/ m²;: on day 1 every 21 days for up to 4 cycles. | 20 months ;Median : 7.5 months |
| 2013Sun, Y. | Advanced NSCLC | 3 | O | 107 | Pemetrexed | pemetrexed 500 mg/ m² | 45 months |
|  |  |  |  | 104 | Docetaxel | docetaxel 75 mg/ m² | 45 months |
| 2012Ardizzoni, A. | Advanced NSCLC | 2 | O | 120 | Pemetrexed | pemetrexed :500 mg/ m²; on day 1 of a 21-day cycle | 42 months  Median :22.2 months (IQR, 15.7 to 28.6) |
|  |  |  |  | 119 | Pemetrexed + carboplatin | carboplatin: AUC 5 mg/ml · min  pemetrexed: 500 mg/ m²; on day 1 every 21 days for up to 4 cycles. | 42 months  Median :22.2 months (IQR, 15.7 to 28.6) |

**D: double-blinded; O: open-labeled**

**Supplementary table S2. (continued)**

| **Study** | **Type** | **Phase** | **Allocation** | **Sample size** | **intervention** | **dose** | **Follow-up** |
| --- | --- | --- | --- | --- | --- | --- | --- |
| 2009 Smit EF  NVALT7 trial | Advanced NSCLC | 2 | O | 121 | Pemetrexed | pemetrexed :500 mg/ m²  course :on day 1 of a 21-day cycle | 12 months  Median:14.7 months( 13.5 to 16.9) |
|  |  |  |  | 119 | Pemetrexed + carboplatin | carboplatin : AUC 5 mg/ml · min pemetrexed: 500 mg/ m²; on day 1 every 21 days for up to 4 cycles. | 12 months  Median:14.7 months( 13.5 to 16.9) |
| Belvedere 2011 | Advanced NSCLC | 2 | O | 25 | Oxaliplatin + docetaxel | Docetaxel (75 mg/ m² on day 1)  Oxaliplatin (70 mg/ m² on day 2) every 3 weeks | 32.2 months |
|  |  |  |  | 25 | Docetaxel | Docetaxel (75 mg/ m²) on day 1 | 22.4 months |
| Pallis, A. G2010. | Advanced NSCLC | 3 | O | 67 | Carboplatin + docetaxel | Docetaxel :50 mg/ m²  Carboplatin: iv, AUC 4 mg/ml | 50 months  Median: 28.0 months (min-max: 20.3-35.7) |
|  |  |  |  | 65 | Docetaxel | Docetaxel :50 mg/ m² | 50 months  Median: 34.5 months (min-max:22.1-46.9) |
| Rebecca 2014 | Advanced NSCLC | 2 | O | 41 | Sunitinib +Pemetrexed | Sunitinib: 37.5 mg daily  Pemetrexed : 500 mg/ m² on day 1 | 50 months |
|  |  |  |  | 42 | Pemetrexed | Pemetrexed : 500 mg/ m² on day 1 | 50 months |
| Nasser 2016 | Advanced NSCLC | 3 | D | 353 | Nintedanib + Pemetrexed | Nintedanib: 200 mg orally twice daily  Pemetrexed : 500 mg/ m² on day1 | 36 months |
|  |  |  |  | 360 | Pemetrexed | Pemetrexed : 500 mg/ m² on Day1 | 36 months |

**D: double-blinded; O: open-labeled**

Supplementary table S3. Indirect comparison for progression free survival in first line therapy.

| Axitinib+PLA | 1.49(0.79,2.81) | 1.09(0.56,2.13) | 0.82(0.4,1.7) | 1.89(0.89,4.02) | 1.79(0.75,4.28) | **0.44(0.22,0.86) *** | 0.95(0.52,1.72) | 1.26(0.58,2.76) | 1.03(0.54,1.96) | 1.25(0.56,2.76) |
| --- | --- | --- | --- | --- | --- | --- | --- | --- | --- | --- |
|  | Bevacizumab+PLA | 0.73(0.51,1.05) | **0.55(0.35,0.88)*** | 1.27(0.77,2.1) | 1.2(0.61,2.35) | **0.29(0.2,0.43) *** | **0.64(0.52,0.78) *** | 0.85(0.49,1.47) | **0.69(0.49,0.96) *** | 0.84(0.47,1.47) |
|  |  | Ipilimumab+PLA | 0.76(0.45,1.26) | **1.74(1.01,3.01) *** | 1.64(0.81,3.32) | **0.4(0.26,0.63) *** | 0.87(0.64,1.17) | 1.16(0.64,2.09) | 0.94(0.63,1.4) | 1.14(0.62,2.1) |
|  |  |  | Nivolumab | **2.3(1.24,4.26) *** | **2.17(1.01,4.64) *** | **0.53(0.31,0.9) *** | 1.15(0.76,1.74) | 1.53(0.8,2.95) | 1.25(0.76,2.03) | 1.51(0.77,2.96) |
|  |  |  |  | Pembrolizumab | 0.94(0.43,2.07) | **0.23(0.13,0.4) *** | **0.5(0.32,0.79) *** | 0.67(0.34,1.32) | **0.54(0.32,0.91) *** | 0.66(0.33,1.32) |
|  |  |  |  |  | Pembrolizumab+PLA | **0.24(0.12,0.5) *** | 0.53(0.28,1) | 0.71(0.31,1.6) | 0.57(0.29,1.14) | 0.7(0.3,1.6) |
|  |  |  |  |  |  | Pemetrexed | **2.17(1.56,3.01) *** | **2.89(1.58,5.29) *** | **2.35(1.55,3.56) *** | **2.86(1.53,5.31) *** |
|  |  |  |  |  |  |  | PLA | 1.33(0.8,2.21) | 1.08(0.84,1.4) | 1.32(0.78,2.23) |
|  |  |  |  |  |  |  |  | Ramucirumab+PLA | 0.81(0.46,1.43) | 0.99(0.47,2.05) |
|  |  |  |  |  |  |  |  |  | Sorafenib+PLA | 1.22(0.68,2.19) |
|  |  |  |  |  |  |  |  |  |  | Vandetanib+PLA |

The hazard ratio (HR) with 95% confidence interval for a given comparison was read in the intersection of two treatments. **P* < 0.05. PLA: doublet platinum-based treatment.

Supplementary table S4. Indirect comparison for progression free survival in subsequent therapy.

| **Aflibercept+Docetaxel** | 0.87(0.55,1.36) | 0.82(0.57,1.17) | 1.04(0.62,1.72) | 1.02(0.57,1.8) | 1.06(0.67,1.66) | 0.93(0.56,1.56) | 0.85(0.55,1.31) | 1.05(0.67,1.65) | 1.11(0.71,1.74) | 0.66(0.34,1.26) | 1.09(0.68,1.74) |
| --- | --- | --- | --- | --- | --- | --- | --- | --- | --- | --- | --- |
|  | **Atezolizumab** | 0.95(0.72,1.24) | 1.2(0.76,1.89) | 1.17(0.69,1.99) | 1.22(0.82,1.81) | 1.07(0.68,1.71) | 0.98(0.68,1.42) | 1.21(0.82,1.79) | 1.28(0.87,1.9) | 0.76(0.41,1.4) | 1.26(0.84,1.89) |
|  |  | **Docetaxel** | 1.27(0.88,1.82) | 1.24(0.79,1.94) | 1.29(0.97,1.71) | 1.14(0.78,1.65) | 1.04(0.81,1.33) | 1.28(0.98,1.69) | **1.35(1.02,1.79)*** | 0.8(0.46,1.39) | 1.33(0.98,1.8) |
|  |  |  | **Nintedanib+Docetaxel** | 0.98(0.55,1.74) | 1.02(0.64,1.61) | 0.9(0.53,1.51) | 0.82(0.53,1.27) | 1.01(0.64,1.6) | 1.07(0.68,1.69) | 0.63(0.33,1.22) | 1.05(0.66,1.69) |
|  |  |  |  | **Nintedanib+Pemetrexed** | 1.04(0.61,1.77) | 0.92(0.51,1.64) | 0.84(0.58,1.22) | 1.04(0.66,1.62) | 1.09(0.64,1.86) | 0.65(0.35,1.2) | 1.07(0.63,1.85) |
|  |  |  |  |  | **Nivolumab** | 0.88(0.55,1.4) | 0.81(0.55,1.17) | 1(0.67,1.47) | 1.05(0.71,1.56) | 0.62(0.33,1.15) | 1.03(0.68,1.56) |
|  |  |  |  |  |  | **Pembrolizumab** | 0.92(0.59,1.43) | 1.13(0.71,1.79) | 1.19(0.75,1.9) | 0.7(0.36,1.37) | 1.17(0.73,1.89) |
|  |  |  |  |  |  |  | **Pemetrexed** | 1.23(0.96,1.58) | 1.3(0.9,1.89) | 0.77(0.47,1.26) | 1.28(0.87,1.89) |
|  |  |  |  |  |  |  |  | **PLA** | 1.05(0.71,1.56) | 0.62(0.36,1.08) | 1.04(0.69,1.56) |
|  |  |  |  |  |  |  |  |  | **Ramucirumab+Docetaxel** | 0.59(0.32,1.1) | 0.98(0.65,1.48) |
|  |  |  |  |  |  |  |  |  |  | **Sunitinib+Pemetrexed** | 1.66(0.89,3.12) |
|  |  |  |  |  |  |  |  |  |  |  | **Vandetanib+Docetaxel** |

The hazard ratio (HR) with 95% confidence interval for a given comparison was read in the intersection of two treatments. **P* < 0.05. PLA: doublet platinum-based treatment. DOCE: docetaxel; PEM: pemetrexed.

Supplementary table S5. Sensitive analysis for efficacy outcome.

| **Removed study** | **Intervention** | **Comparator** |
| --- | --- | --- |
| **Overall survival** | | |
| **First-line therapy (**PLA : doublet platinum-based treatment) | | |
|  | Bevacizumab+PLA | Sorafenib+PLA |
| None | **HR : 0.82(0.66,1.01)** | |
| 2010Reck, M. | HR : 0.75(0.62,0.9) | |
|  | Bevacizumab+PLA | PLA |
| None | **HR : 0.86(0.75,0.99)** | |
| 2011Wang, Y. | HR : 0.86(0.75,1) | |
| 2012Lynch TJ | HR : 0.86(0.74,1) | |
| 2013Zukin, M | HR : 0.86(0.74,1) | |
| 2012Rogerio L.(Abstract) | HR : 0.86(0.74,1) | |
|  | Pemetrexed | Ramucirumab+PLA |
| None | **HR : 1.63(1.01,2.62)** | |
| 2012Lynch TJ | HR : 1.63(0.99,2.67) | |
| NCT01285609 | HR : 1.63(0.99,2.67) | |
| 2013Zukin, M | HR : 1.7(0.97,2.96) | |
| 2012Rogerio L. (Abstract) | HR : 1.56(0.9,2.71) | |
|  | Nivolumab | Pemetrexed |
| None | HR :**0.61(0.42,0.88)** | |
| 2012Rogerio L.(Abstract) | HR : 0.63(0.4,1) | |
| **Subsequent therapy** |  |  |
|  | Nivolumab | PLA |
| None | **HR : 0.78(0.63,0.97)** | |
| 2011Belvedere | HR : 0.83(0.65,1.06) | |
| 2012Ardizzoni, A. | HR : 0.8(0.64,1) | |
|  | Ramucirumab+Docetaxel | Sunitinib+Pemetrexed |
| None | **HR : 0.62(0.4,0.97)** | |
| 2011Belvedere | HR : 0.63(0.4,1) | |
|  | Docetaxel | PLA |
| None | **HR : 1.15(1,1.32)** | |
| 2012Ardizzoni, A. | HR : 1.17(1.01,1.35) | |
| 2011Belvedere | HR :1.21(1.01,1.46) | |
|  | PLA | Sunitinib+Pemetrexed |
| None | **HR : 0.63(0.41,0.97)** | |
| 2009 Smit EF | HR :0.64(0.41,1) | |

**Supplementary table S5. (continued)**

| **Removed study** | **Intervention** | **Comparator** |
| --- | --- | --- |
| **Progression free survival** | | |
| **First line therapy** |  |  |
|  | Ipilimumab+PLA | Pembrolizumab |
| None | **HR : 1.74(1.01,3.01)** | |
| 2012Niho, S. | HR : 1.74(0.99,3.06) | |
| 2011Wang, Y. | HR : 1.74(1,3.03) | |
| 2010Scagliotti, G. | HR : 1.74(0.96,3.16) | |
| 2012Lynch TJ | HR : 1.74(0.93,3.24) | |
| NCT01285609 | HR : 1.74(0.85,3.55) | |
| 2013Zukin, M | HR : 1.74(0.98,3.08) | |
| 2012Rogerio L.(Abstract) | HR : 1.74(0.98,3.08) | |
|  | Pembrolizumab+PLA | PLA |
| None | **HR : 0.5(0.32,0.79)** | |
| 2011Wang, Y. | HR : 0.53(0.28,1.01) | |
| 2014 Belani CP | HR : 0.53(0.28,1) | |
|  | Nivolumab | Pembrolizumab+PLA |
| None | **HR : 2.17(1.01,4.64)** | |
| 2012Lynch TJ | HR : 2.17(0.99,4.77) | |
| NCT01285609 | HR : 2.17(0.99,4.77) | |
| 2013Zukin, M | HR : 2.17(0.99,4.75) | |
| 2012Niho, S. | HR : 2.17(1,4.72) | |
|  | Nivolumab | Pemetrexed |
| None | **HR : 0.53(0.31,0.9)** | |
| 2013Zukin, M | HR : 0.53(0.28,1.02) | |
|  | Bevacizumab+PLA | Ipilimumab+PLA |
| None | **HR : 0.73(0.51,1.05)** | |
| 2010Reck, M. | HR : 0.65(0.48,0.89) | |
|  | Bevacizumab+PLA | Sorafenib+PLA |
| None | **HR : 0.69(0.49,0.96)** | |
| 2010Scagliotti, G. | HR : 0.72(0.46,1.13) | |

**Supplementary table S5. (continued)**

| **Removed study** | **Intervention** | **Comparator** |
| --- | --- | --- |
| **Progression free survival** | | |
| **Subsequent therapy** |  |  |
|  | Pemetrexed | PLA |
| NONE | HR : 1.23(0.97,1.57) | |
| 2012Ardizzoni, A. | HR : 1.42(1.08,1.86) | |
|  | Docetaxel | PLA |
| NONE | HR : 1.28(0.98,1.69) | |
| 2012Ardizzoni, A. | HR : 1.4(1.06,1.83) | |
|  | PLA | Sunitinib+Pemetrexed |
| NONE | HR :0.62(0.36,1.08) | |
| 2012Ardizzoni, A. | HR :0.54(0.32,0.93) | |
|  | Ramucirumab+Docetaxel | Sunitinib+Pemetrexed |
| NONE | HR :0.59(0.32,1.1) | |
| 2012Ardizzoni, A. | HR :0.56(0.32,0.99) | |
| 2011Belvedere | HR :0.55(0.32,0.95) | |
|  | Pemetrexed | Ramucirumab+Docetaxel |
| NONE | HR :1.3(0.9,1.89) | |
| 2011Belvedere | HR :1.39(1.02,1.91) | |
|  | Sunitinib+Pemetrexed | Vandetanib+Docetaxel |
| NONE | HR :1.66(0.89,3.12) | |
| 2011Belvedere | HR :1.77(1.02,3.06) | |

**
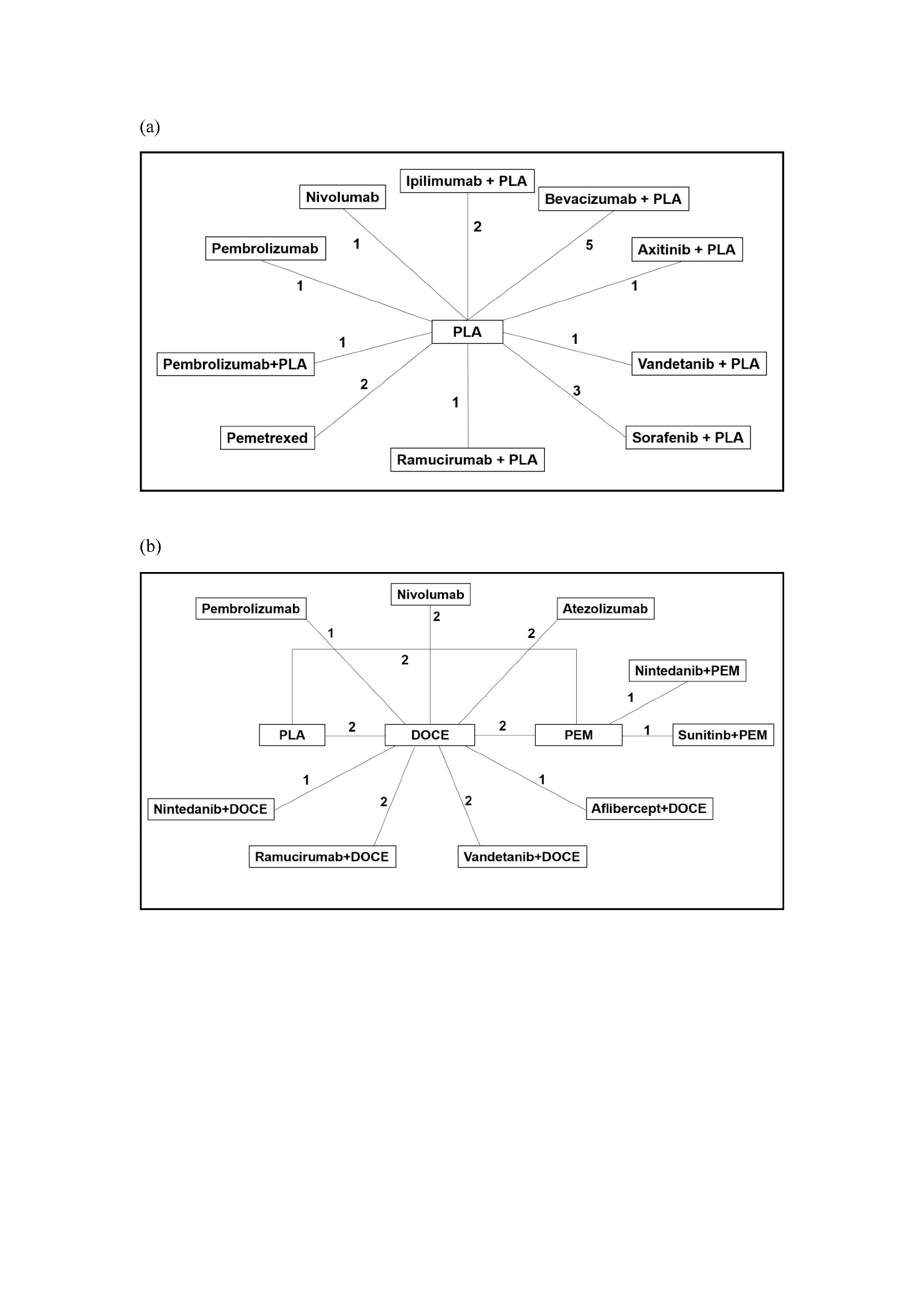
**

**Supplementary figure S1. Comparison plot for (a) first line therapy and (b) subsequent therapy.**

The solid line represents as the direct comparisons and the number reflected the number of trials. PLA: doublet platinum-based treatment; PEM: pemetrexed; DOCE: docetaxel.


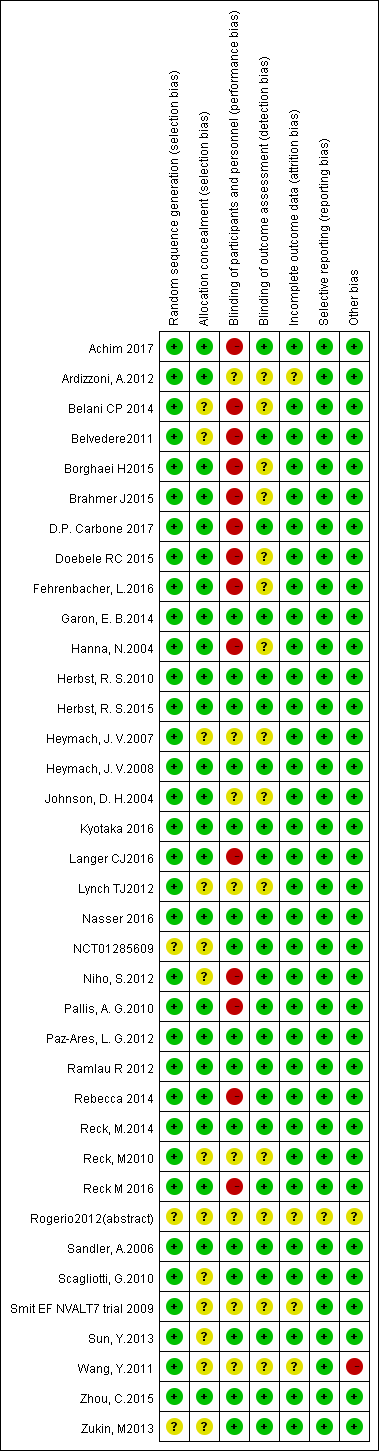

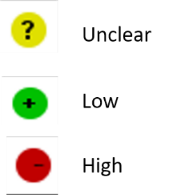

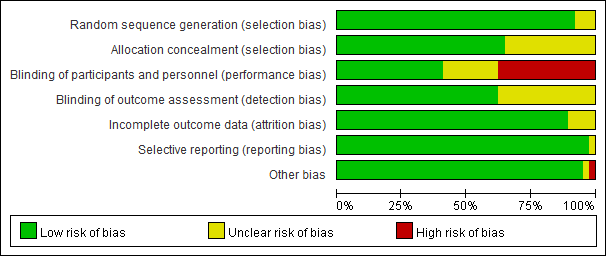


# Supplementary figure S2. Risk of bias

(a) Risk of bias summary: The judgements about each risk of bias item for each included study. (b) Risk of bias graph: The judgements about each risk of bias item reflected as percentages across all included studies.


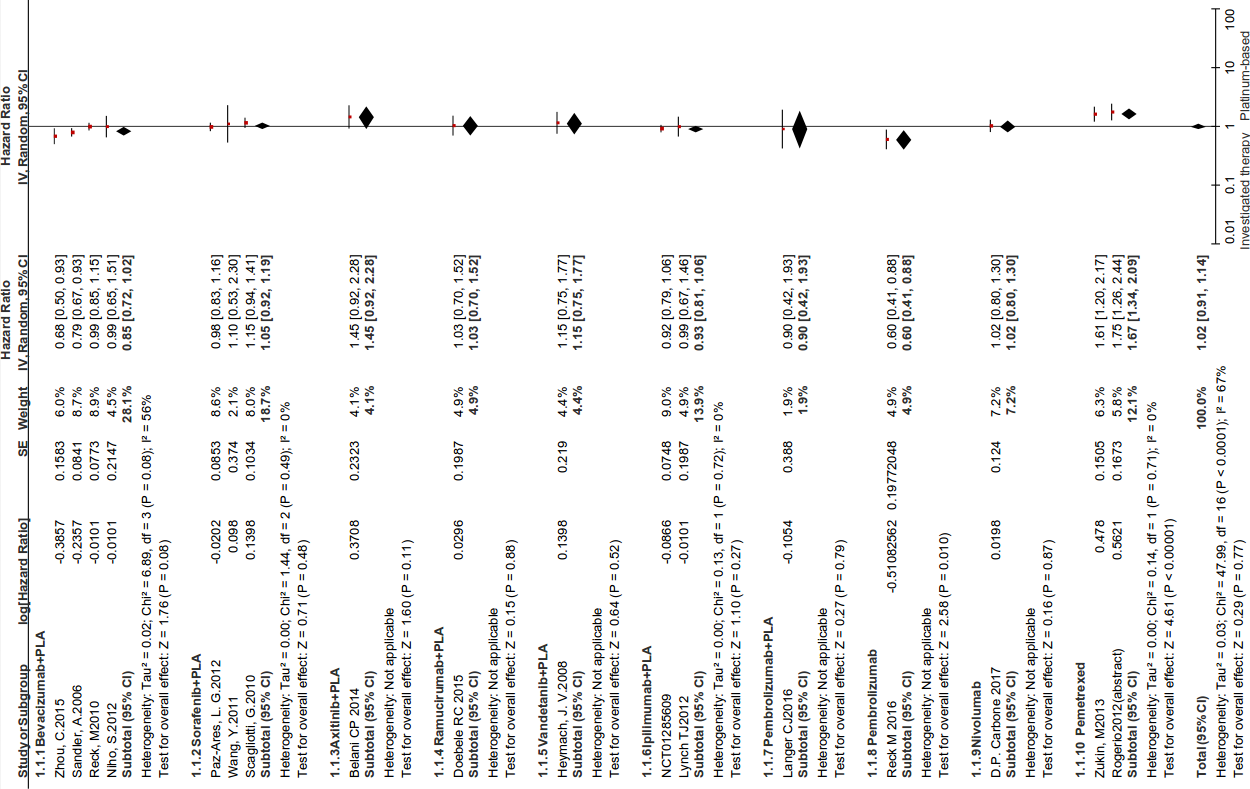


Supplementary figure S3. Pairwise comparisons of overall survival in first line therapy: investigated therapy vs. doublet platinum-based therapy.

Data presented as hazard ratio (HR) with 95% confidence interval (CI); p < 0.05: statistically significance. Square represented estimated HR in each study and its size reflected the sample size; 95% CIs represented horizon lines; summary HR presented as diamond. The significant level of heterogeneity was p < 0.1.


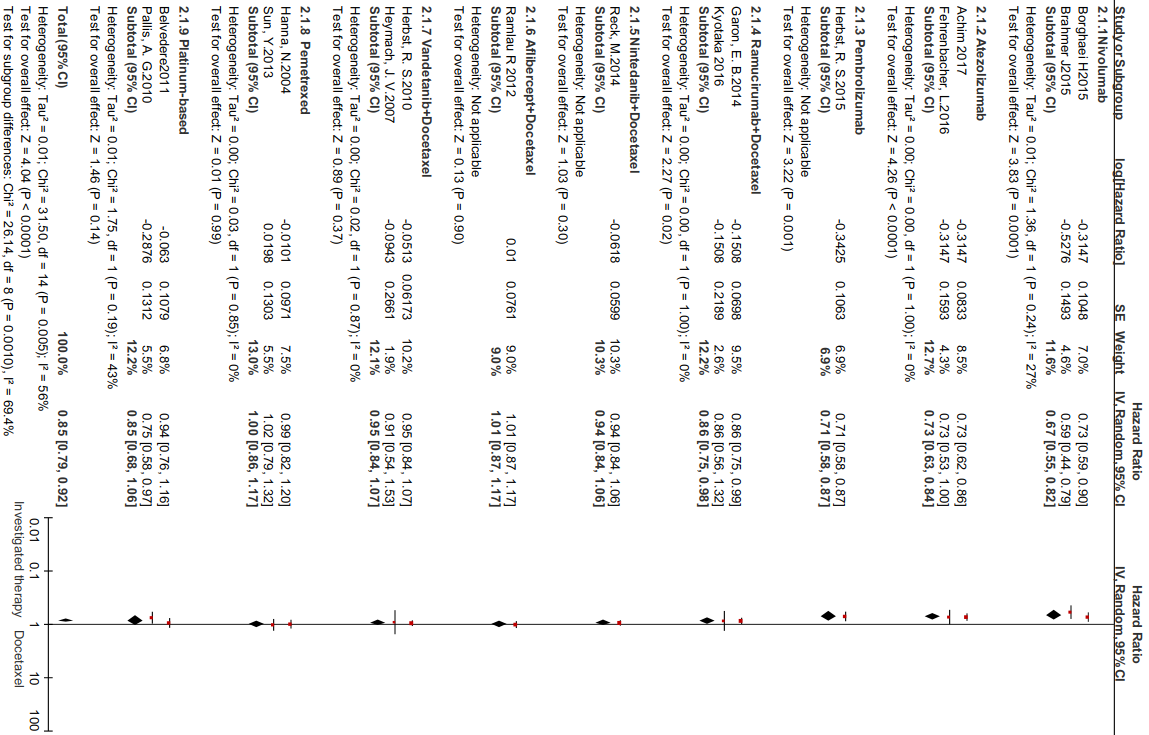


Supplementary figure S4. Pairwise comparisons of overall survival in subsequent therapy: investigated therapy vs. docetaxel.

Data presented as hazard ratio (HR) with 95% confidence interval (CI); p < 0.05: statistically significance. Square represented estimated HR in each study and its size reflected the sample size; 95% CIs represented horizon lines; summary HR presented as diamond. The significant level of heterogeneity was p < 0.1.


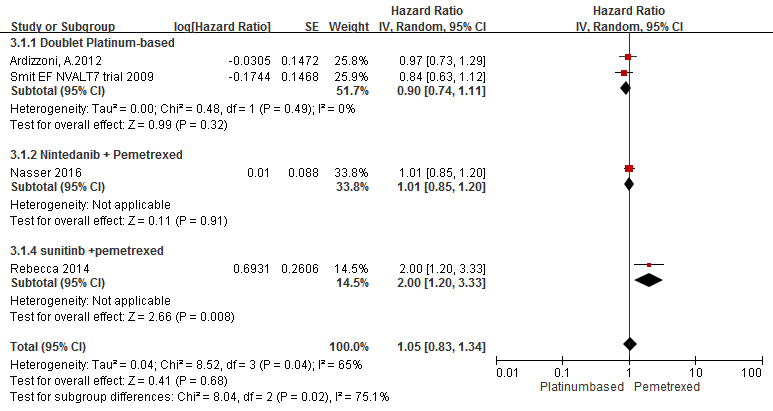


Supplementary figure S5. Pairwise comparisons of overall survival in subsequent therapy: investigated therapy vs. pemetrexed.

Data presented as hazard ratio (HR) with 95% confidence interval (CI); p < 0.05: statistically significance. Square represented estimated HR in each study and its size reflected the sample size; 95% CIs represented horizon lines; summary HR presented as diamond. The significant level of heterogeneity was p < 0.1.


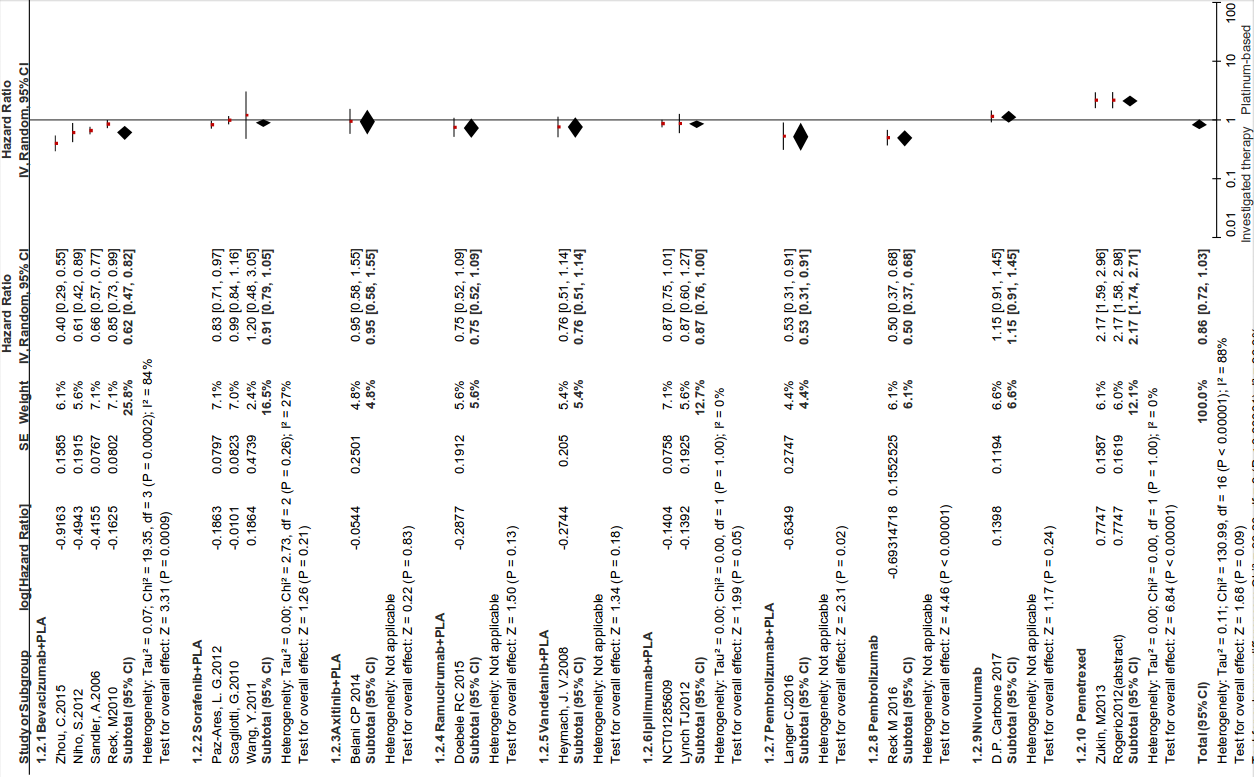


Supplementary figure S6. Pairwise comparisons of progression free survival in first line therapy: investigated therapy vs. doublet platinum-based treatment.

Data presented as hazard ratio (HR) with 95% confidence interval (CI); p < 0.05: statistically significance. Square represented estimated HR in each study and its size reflected the sample size; 95% CIs represented horizon lines; summary HR presented as diamond. The significant level of heterogeneity was p < 0.1.


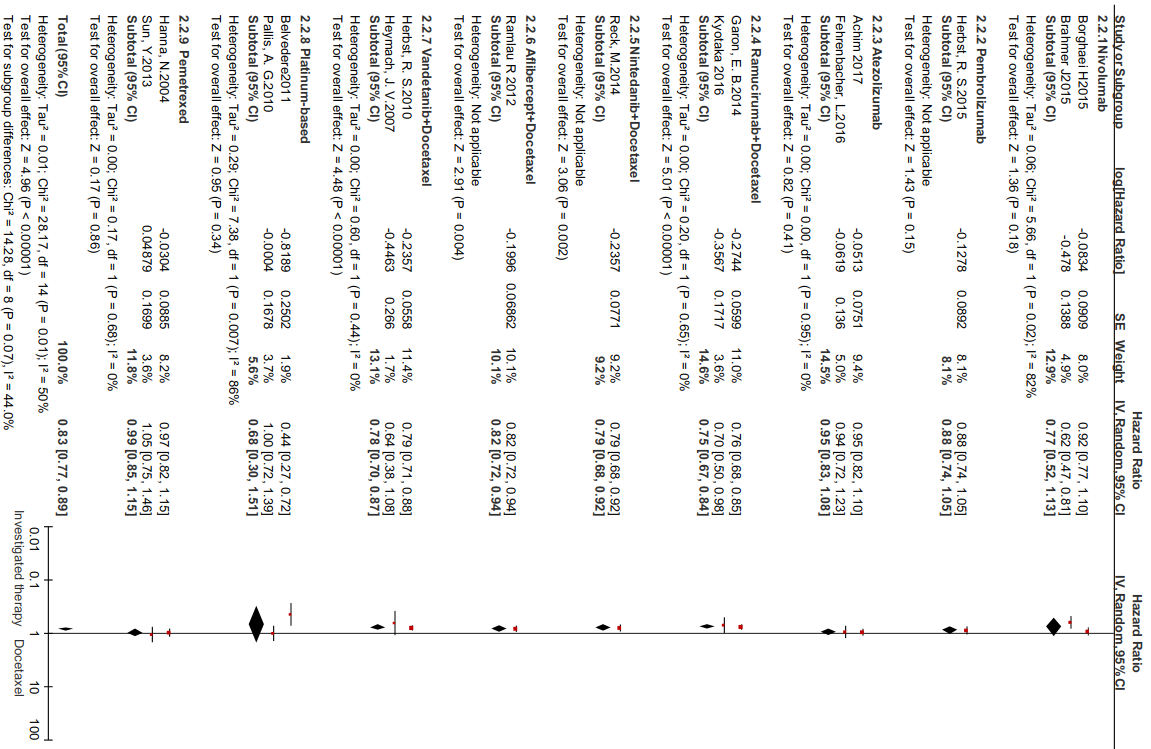


Supplementary figure S7. Pairwise comparisons of progression free survival in subsequent therapy: investigated therapy vs. docetaxel.

Data presented as hazard ratio (HR) with 95% confidence interval (CI); p < 0.05: statistically significance. Square represented estimated HR in each study and its size reflected the sample size; 95% CIs represented horizon lines; summary HR presented as diamond. The significant level of heterogeneity was p < 0.1.


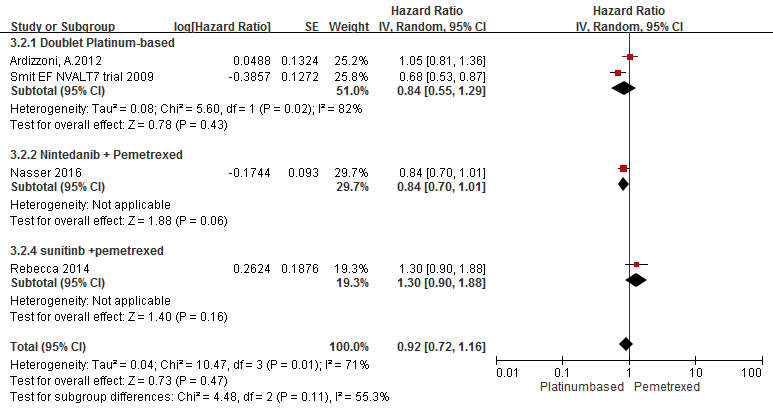


Supplementary figure S8. Pairwise comparisons of progression free survival in subsequent therapy: investigated therapy vs. pemetrexed.

Data presented as hazard ratio (HR) with 95% confidence interval (CI); p < 0.05: statistically significance. Square represented estimated HR in each study and its size reflected the sample size; 95% CIs represented horizon lines; summary HR presented as diamond. The significant level of heterogeneity was p < 0.1.


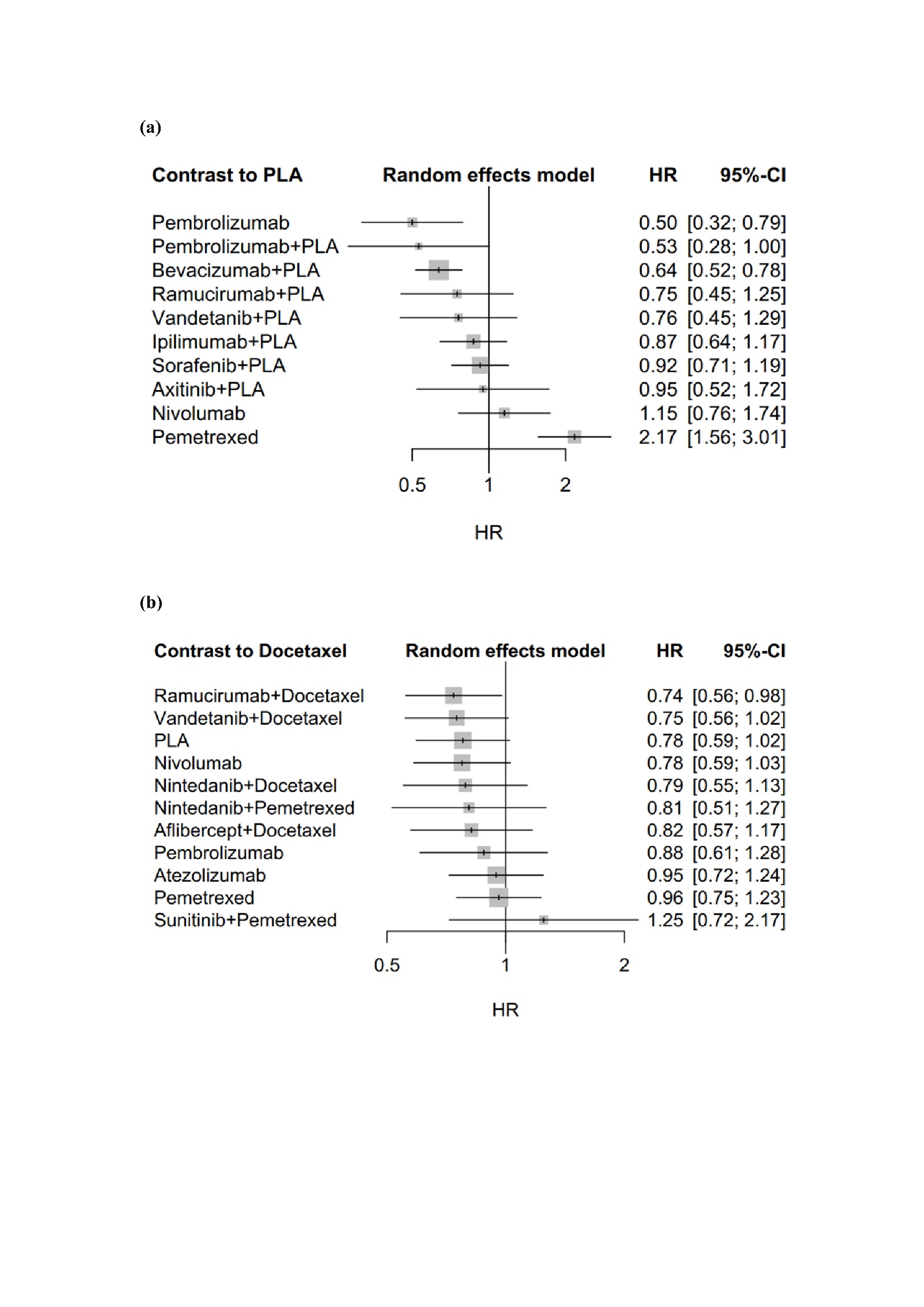


**Supplementary figure S9. Forest plot of indirect meta-analysis: Progression free survival in (a) first line therapy and (b) subsequent therapy.** All individual regimens compared with reference treatment. Doublet platinum-based treatment was the reference treatment in the first line therapy and docetaxel was the reference treatment in the subsequent therapy. Hazard ratios (HR) and 95% confidence intervals were given. PLA: doublet platinum-based treatment.


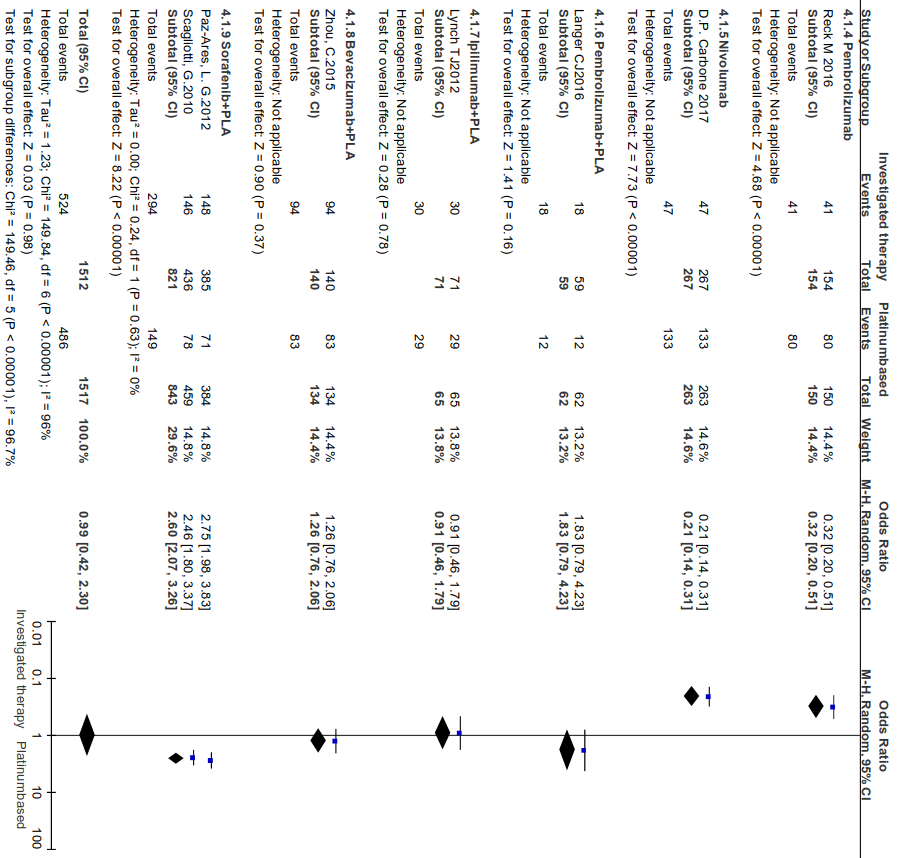


Supplementary figure S10. Pairwise comparisons of all grade 3 to 5 adverse events: investigated therapy vs. doublet platinum-based treatment.

Data presented as odds ratio (OR) with 95% confidence interval (CI); p < 0.05: statistically significance. Square represented estimated OR in each study and its size reflected the sample size; 95% CIs represented horizon lines; summary OR presented as diamond. The significant level of heterogeneity was p < 0.1.


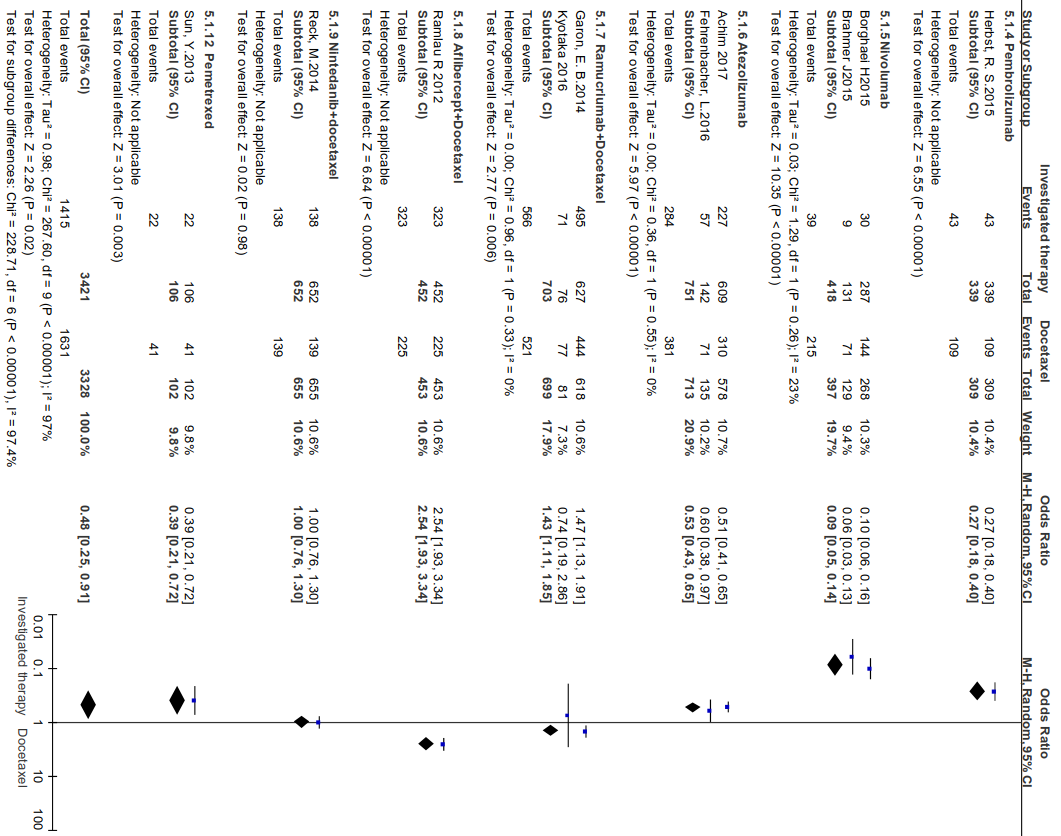


Supplementary figure S11. Pairwise comparisons of all grade 3 to 5 adverse events: investigated therapy vs. docetaxel.

Data presented as odds ratio (OR) with 95% confidence interval (CI); p < 0.05: statistically significance. Square represented estimated OR in each study and its size reflected the sample size; 95% CIs represented horizon lines; summary OR presented as diamond. The significant level of heterogeneity was p < 0.1.


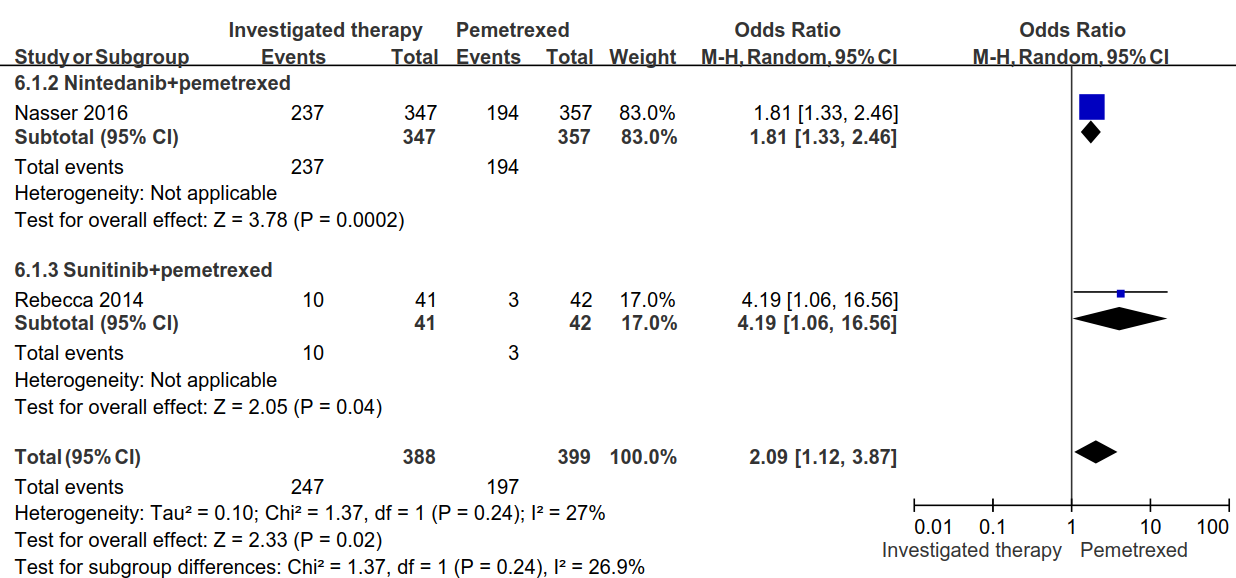


Supplementary figure S12. Pairwise comparisons of all grade 3 to 5 adverse events: investigated therapy vs. pemetrexed.

Data presented as odds ratio (OR) with 95% confidence interval (CI); p < 0.05: statistically significance. Square represented estimated OR in each study and its size reflected the sample size; 95% CIs represented horizon lines; summary OR presented as diamond. The significant level of heterogeneity was p < 0.1.
